# Supplementary figures and images for: Differential adhesion during development establishes individual neural stem cell niches and shapes adult behaviour in Drosophila
Source: PLoS Biol. 2023 Nov 9;21(11):e3002352. doi: 10.1371/journal.pbio.3002352 (PMC10635556; doi:10.1371/journal.pbio.3002352)

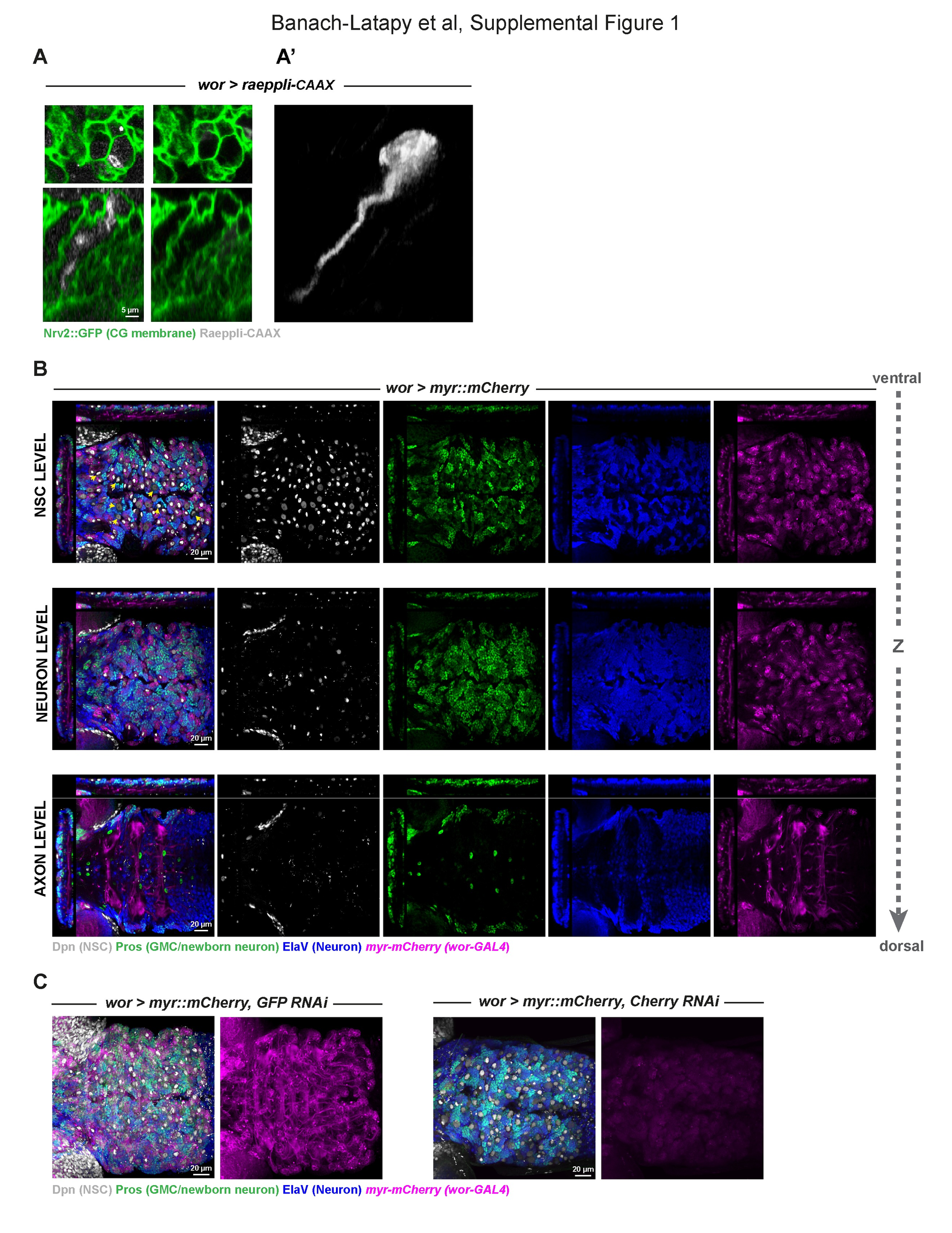

Supplement: S1 Fig — (A) Confocal picture of one NSC lineage labelled with a membrane marker (mTFP1-CAAX, grey). NSC lineages are marked with the multicolour lineage tracing Raeppli-CAAX (blue, white, orange, and red) under the control of the GAL/UAS system and induced at ALH0 using hs-Flp (wor > raeppli-CAAX). Larvae are dissected after 68 h at 29°C from ALH0. One mTFP1+ clone is shown (n > 45 clones). CG membrane was visualised with Nrv2::GFP (green). (A’) 3D reconstruction of the membrane signal of the NSC lineage shown in (A). (B) Representative confocal pictures of the expression pattern of worniu-GAL4 (wor >) along the dorso-ventral axis of the VNC, at the NSC level, at the neuron level, and at the axon level. UAS-myr-mCherry was driven under the control of wor-GAL4. Larvae are dissected after 72 h at 25°C from ALH0. n = 8 VNCs. worniu-GAL4 expression is visualised by the membrane staining of mCherry (magenta), NSCs are labelled with anti-Dpn (grey), GMC and newborn neurons with anti-Prospero (green), and neurons with anti-ElaV (blue). Of note, we found that wor-GAL4 does not drive in a few Type I NSCs in the VNC (negative for the mCherry signal, yellow arrows). (C) Representative confocal z-projections (maximal intensity) of the expression pattern of worniu-GAL4 (wor >) in control and under RNAi knockdown of mCherry. wor-GAL4, tub-GAL80ts was used to drive from ALH0 UAS-myr-mCherry combined with either UAS-GFP RNAi (control, n = 7 VNCs) or UAS-mCherry RNAi (mCherry knockdown, n = 8 VNCs). Larvae are dissected after 68 h at 29°C from ALH0. worniu-GAL4 expression is visualised by the membrane staining of mCherry (magenta), NSCs are labelled with anti-Dpn (grey), GMC and newborn neurons with anti-Prospero (green), and neurons with anti-ElaV (blue). ALH, after larval hatching; CG, cortex glia; GMC, ganglion mother cell; NSC, neural stem cell; VNC, ventral nerve cord. (TIF) [file pbio.3002352.s001.tif]

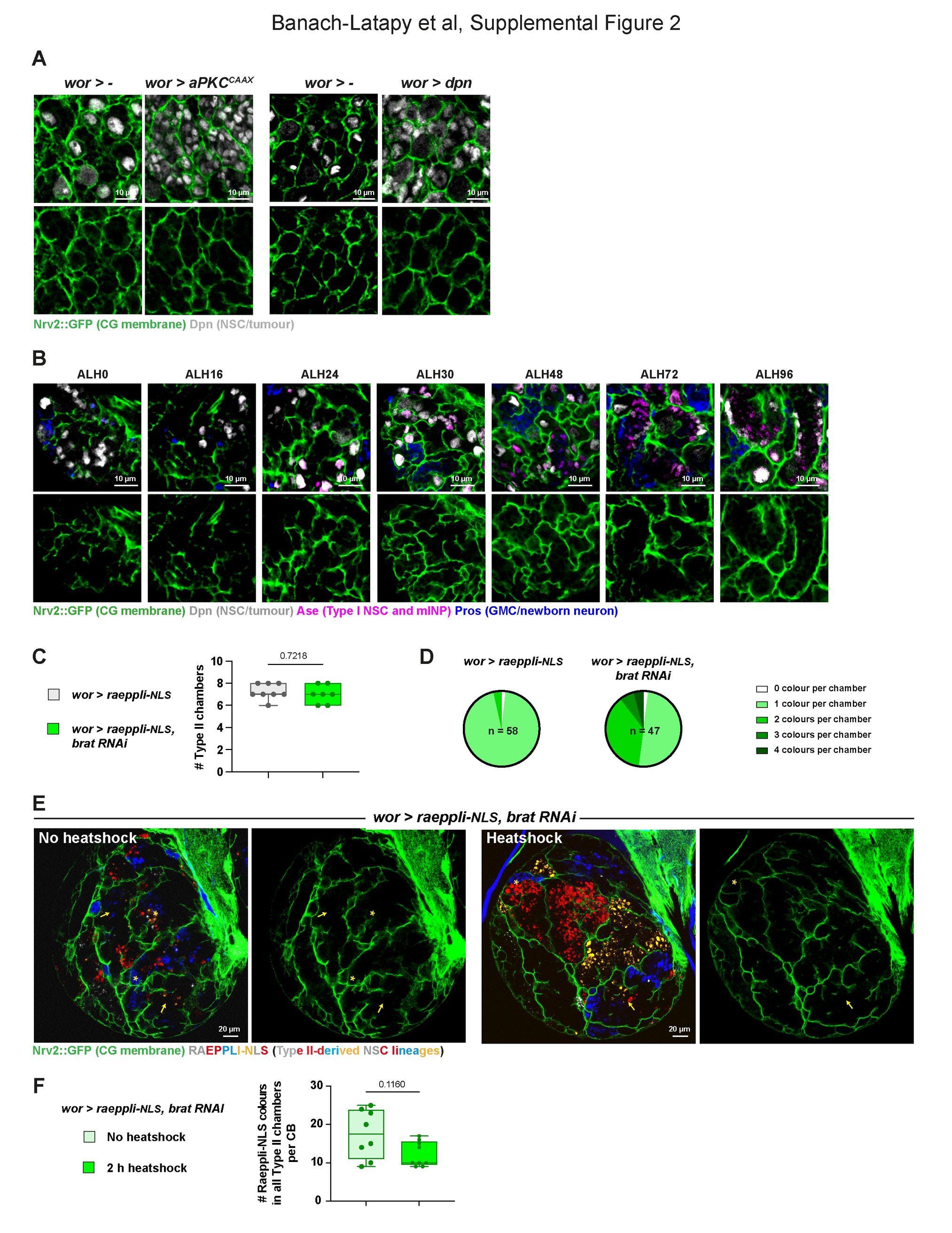

Supplement: S2 Fig — (A) Two different Type I tumours, aPKC (Nrv2::GFP, wor-GAL4; tub-GAL80ts > aPKCCAAX) and dpn (Nrv2::GFP, wor-GAL4; tub-GAL80ts > dpn), were induced from ALH0. aPKC larvae were dissected after 48 h at 18°C, followed by 48–52 h at 29°C. dpn larvae were dissected after 72 h at 29°C. aPKC tumours, n = 6 VNCs and dpn tumours, n = 6 VNCs. The organisation of CG membrane was monitored by Nrv2::GFP (green). NSCs are labelled with anti-Dpn (grey). (B) Representative confocal images of the progressive formation of individual encasing around Type II lineages, at ALH0, ALH16, ALH24, ALH30, ALH48, ALH72, and ALH96 at 25°C. n ≥ 8 CBs for all time points. CG membrane is visualised with Nrv2::GFP (green), NSCs are labelled with anti-Dpn (grey), Type II NSCs are recognised by the absence of anti-Ase (magenta), and GMCs are labelled with anti-Pros (blue). (C) Quantification of the number of CG chambers containing Type II-derived lineages, in control (wor > raeppli-NLS) and brat RNAi (wor > raeppli-NLS, brat RNAi), from Fig 2D. Briefly, Type II (brat RNAi) tumours were induced together with the multicolour lineage tracing Raeppli-NLS. One out of 4 colours (blue, white, orange, and red) is stochastically expressed in the transformed NSCs upon induction. Larvae were dissected after 48 h at 18°C, followed by 2 h heatshock at 37°C and 48–52 h at 29°C. See S1 Table for detailed genetics, timing, and conditions of larval rearing. Control (n = 8 CB) and brat RNAi (n = 7 CB). Data statistics: Mann–Whitney U test. Results are presented as box and whisker plots. (D) Quantification of the number of colours per chambers containing Type II-derived lineages in control and brat RNAi, from (C). Several colours can be found in one CG chamber for control Type II and especially Type II brat tumours, while average chamber number did not change (see (C)). An explanation is the non-controlled activation of Raeppli-NLS in tumour NSCs/INPs in which the original heatshock-induced recombination failed. This wo [file pbio.3002352.s002.tif]

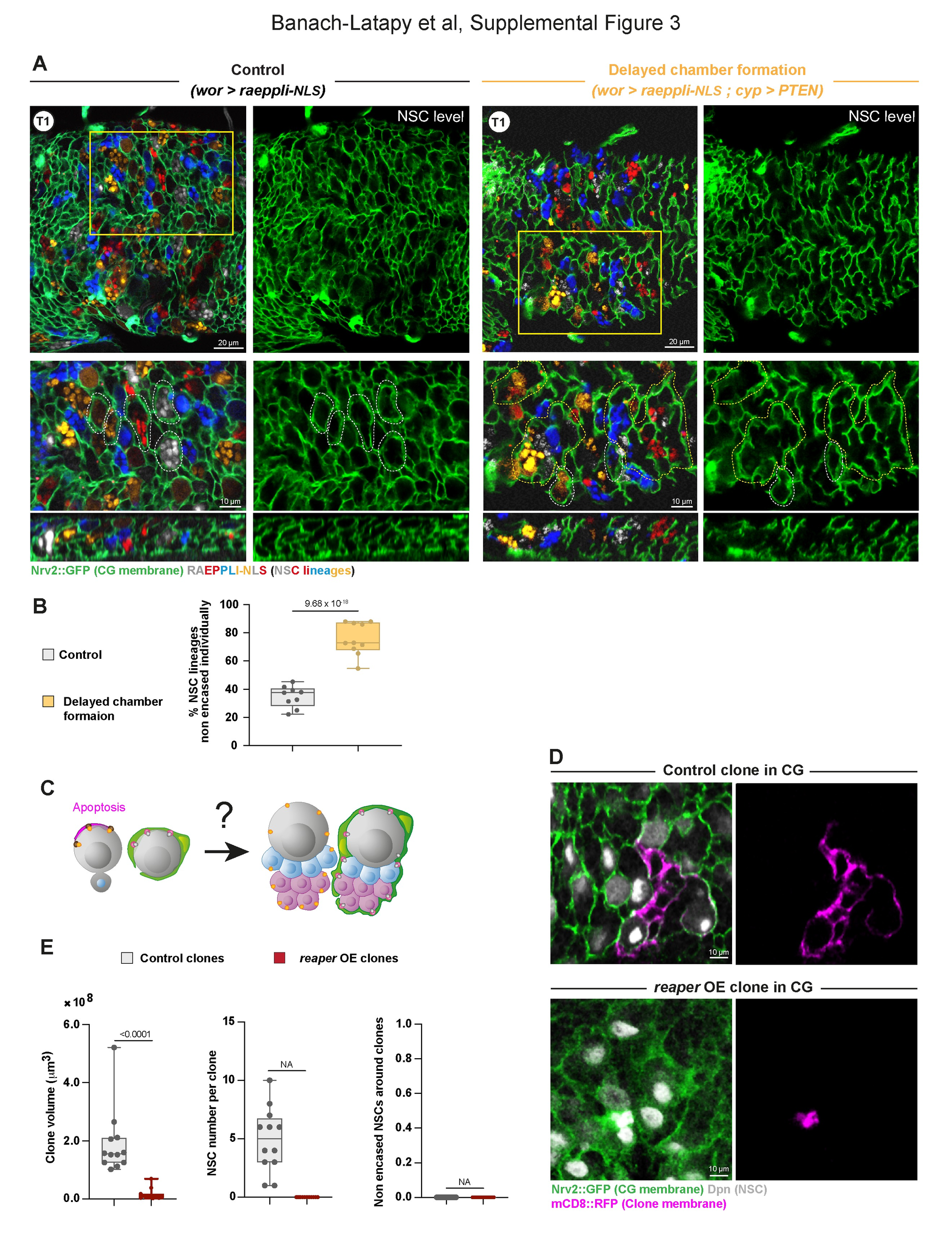

Supplement: S3 Fig — (A) Representative confocal pictures of the extent of individual encasing of NSC lineages by CG at T1 both for Control and Delayed chamber formation conditions, following the regimen described in Fig 3A. Top panel shows the whole thoracic VNC, and bottom panel a close-up of the yellow box. NSC lineages were marked with the multicolour lineage tracing Raeppli-NLS (blue, white, orange, and red), induced at ALH0 using hs-Flp (wor > raeppli-NLS). Larvae are dissected after 72 h at 29°C (T1). See S1 Table for detailed genetics, timing, and conditions of larval rearing. CG membrane was visualised with Nrv2::GFP (green). Dashed white lines highlight examples of NSC lineages already encased individually, and dashed yellow lines outline zones where NSC lineages are still not individually encased. (B) Quantification of the percentage of NSC lineages non-individually encased from (A). Control T1 (n = 9 VNCs) and Delayed chamber formation T1 (n = 10 VNCs). Data statistics: generalised linear model (Binomial regression with a Bernoulli distribution). Results are presented as box and whisker plots. (C) Schematic of the experiment designed to probe whether specific adhesions exist between individual CG cells and individual NSC lineages. Mosaic clonal analysis is used to induce apoptosis in a few CG in a random fashion (magenta), preventing the encasing of corresponding NSC lineages. If specific adhesions exist between individual CG cells and individual NSC lineages, neighbouring CG cells would not be able to bind to and encase these lineages. (D) Representative confocal picture of the CG network in control CG clone and in clone where apoptosis has been induced earlier (reaper OE). The CoinFLP system was used to generate wild-type and reaper OE clones in the CG. Clones were induced at late embryogenesis/early larval stage through the expression of cyp4g15-FLP. Larvae were then maintained at 18°C for 96 h to prevent reaper expression and then shifted to 29°C for 48 h and dissected. [file pbio.3002352.s003.tif]

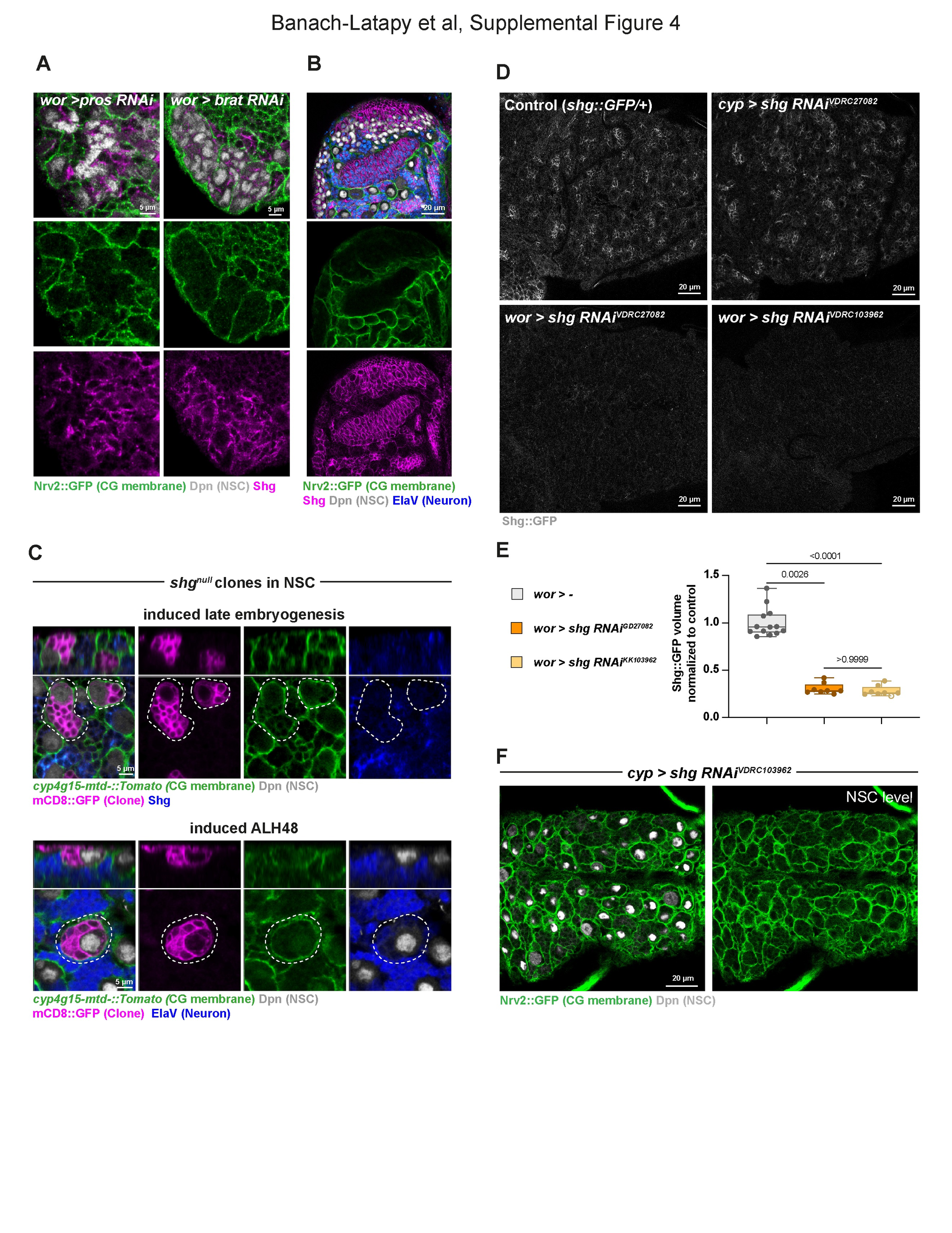

Supplement: S4 Fig — (A) Representative confocal pictures of the expression of Shg in Type I (wor > pros RNAi) and Type II (wor > brat RNAi) tumours from Fig 2A and 2B. pros tumour (n = 10 VNCs) and brat tumour (n = 10 CBs). The organisation of CG membrane was monitored by Nrv2::GFP (green). Tumour NSCs are labelled with anti-Dpn (grey), and Shg is detected with a specific antibody (magenta). (B) Representative confocal image of the expression of Shg in the OL (ALH72 at 25°C). n = 6 OLs. Shg is detected with a specific antibody (magenta), CG membrane is visualised with Nrv2::GFP (green), NSCs are labelled with Dpn (grey), and neurons are labelled with ElaV (blue). (C) Confocal images of mutant clones of shg (null shgR64 allele) in NSC lineages, generated during late embryogenesis (top panel) or at ALH48 at 25°C (bottom panel) by heatshock induction through hs-FLP. Clones are analysed at ALH72 at 25°C. n(embryogenesis) = 59 clones for 11 VNCs and n(ALH48) = 11 clones for 8 VNCs. See S1 Table for detailed genetics, timing, and conditions of larval rearing. The membrane of the clone is marked by mCD8::GFP (magenta). CG membrane is visualised with cyp4g15-mtd::Tomato (green), NSCs are labelled with Dpn (grey), Shg is detected with a specific antibody (blue, induction during embryogenesis), and neurons are labelled with ElaV (blue, induction at ALH48). (D) Representative confocal pictures of the loss of signal for Shg (monitored through Shg::GFP, grey) in different genetic conditions defined by the driver line (CG, cyp4g15-GAL4, cyp >; NSC lineages, wor-GAL4, wor >) and RNAi constructs. Larvae are dissected after 68 h at 29°C from ALH0. (E) Quantification of the efficiency of shg knockdown in NSC lineages for different RNAi lines from (D). Shg levels are monitored by Shg::GFP. See Methods for details of the quantification. Control (shg::GFP/+) (n = 13 VNCs), wor > shg RNAiVDRC27082 (n = 8 VNCs), and wor > shg RNAiVDRC103962 (n = 8 VNCs). Data statistics: Kruskal–Wallis H test with Dunn’s mul [file pbio.3002352.s004.tif]

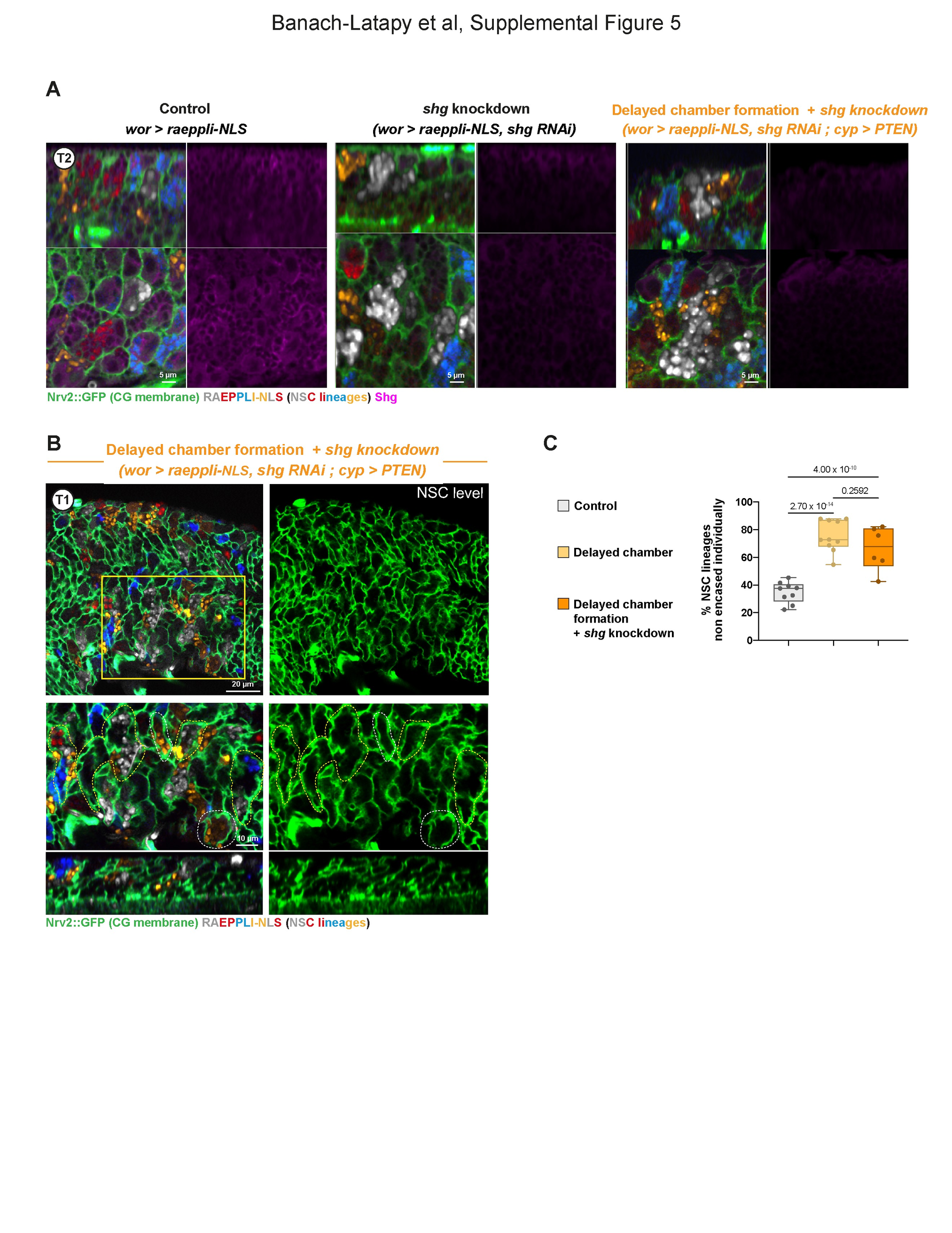

Supplement: S5 Fig — (A) Close-ups of confocal pictures assessing Shg levels in control, shg knockdown (VDRC line 27082) in NSC lineages (Fig 4E) and shg knockdown in NSC lineages plus delayed chamber formation (Fig 5B) at T2 (100 h at 29°C). n ≥ 5 for all conditions. Shg levels are monitored with a specific antibody (magenta), NSC lineages are marked with the multicolour lineage tracing Raeppli-NLS (wor > raeppli-NLS; blue, white, orange, and red), and CG membrane is visualised with Nrv2::GFP (green). See S1 Table for detailed genetics, timing, and conditions of larval rearing. (B) Representative confocal pictures of the extent of individual encasing of NSC lineages by the CG at T1 (72 h at 29°C), following the regimen described in Fig 5A. Top panel shows the whole thoracic VNC, and bottom panel a close-up of the yellow box. NSC lineages were marked with the multicolour lineage tracing Raeppli-NLS (blue, white, orange, and red), induced at ALH0 using hs-Flp (wor > raeppli-NLS). See S1 Table for detailed genetics, timing, and conditions of larval rearing. CG membrane was visualised with Nrv2::GFP (green). Dashed white lines highlight examples of NSC lineages already encased individually, and dashed yellow lines outline zones where NSC lineages are still not individually encased. (C) Quantification of the percentage of NSC lineages non-individually encased at T1 in Control, in shg knockdown (VDRC line 27082) in NSC lineages, and in shg knockdown in NSC lineages plus delayed chamber formation. Control T1 (n = 9 VNCs), Delayed chamber formation T1 (n = 10 VNCs), and Delayed chamber formation + shg RNAi in NSC lineages T1 (n = 6 VNCs). Data statistics: generalised linear model (Binomial regression with a Bernoulli distribution). p = 2.59 × 10^−18^ for the grouped dataset. P values for individual comparisons test are displayed on the graph. Results are presented as box and whisker plots, where whiskers mark the minimum and maximum, the box includes the 25th–75th percentile, and the line in the [file pbio.3002352.s005.tif]

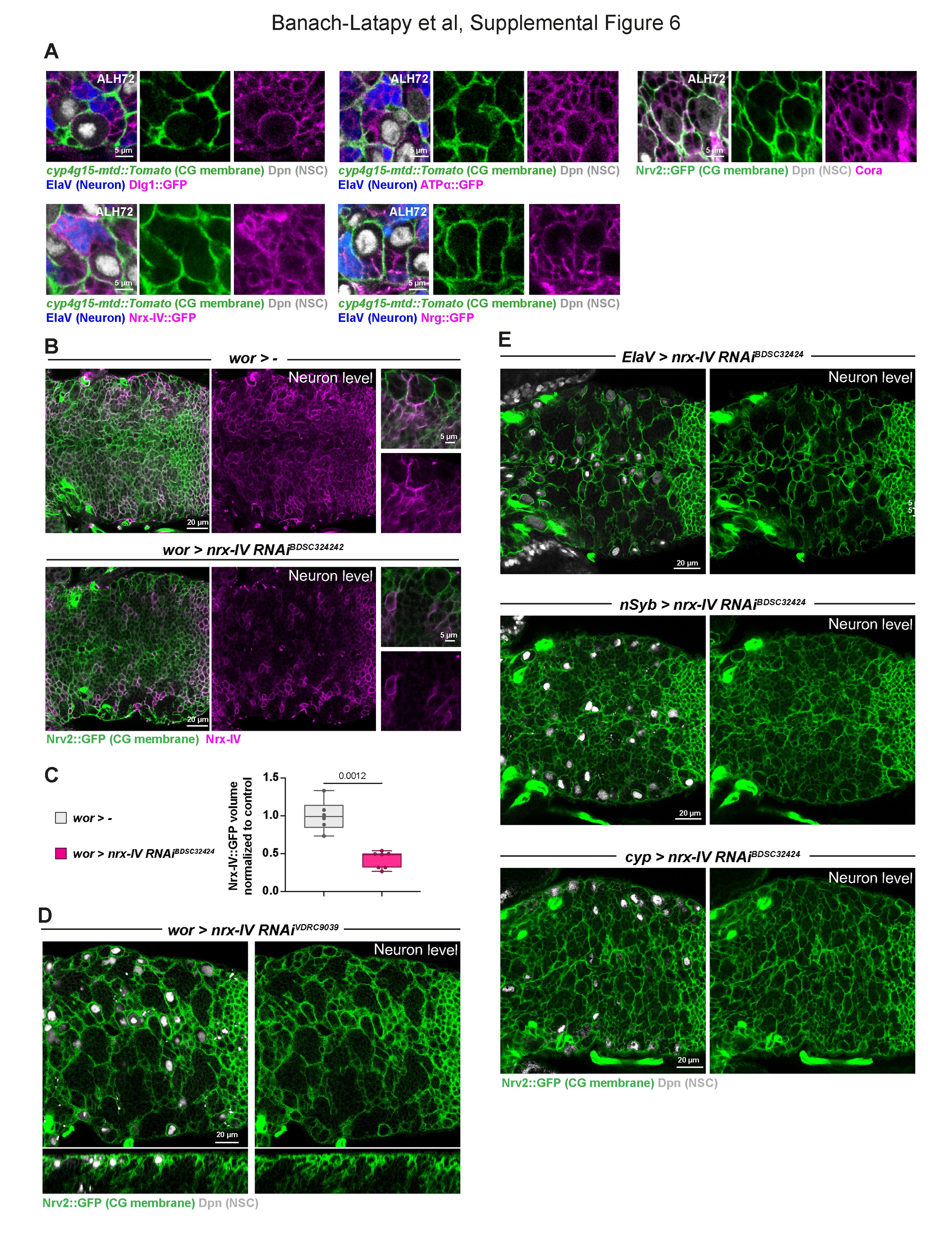

Supplement: S6 Fig — (A) Representative confocal images of the expression of the septate junction components Dlg1, ATPα, Cora, Nrx-IV, and Nrg at ALH72 at 25°C. n ≥ 6 for all components. Dlg1 is monitored through a Dlg1::GFP fusion, ATPα through an ATPα::GFP fusion, Nrx-IV through a Nrx-IV::GFP fusion, Nrg through a Nrg::GFP fusion, and Cora is detected by a specific antibody (all magenta). CG membrane is visualised by cyp4g15-mtd::Tomato (green), NSCs are labelled with anti-Dpn (grey), and neurons are labelled with anti-ElaV (blue). (B) Representative confocal pictures of the thoracic VNC for control (wor >—(x w1118)) and for nrx-IV knockdown by RNAi (wor > nrx-IV RNAi, BDSC line 324242) in NSC lineages (driver line Nrv2::GFP, wor-GAL4; tub-GAL80ts). Larvae are dissected after 68 h at 29°C from ALH0. Nrx-IV levels are monitored by a specific antibody (magenta), and CG membrane is visualised by Nrv2::GFP (green). (C) Quantification of the efficiency of nrx-IV knockdown in NSC lineages by RNAi from (B). Nrx-IV levels are monitored by a specific antibody. See Methods for details of the quantification. wor >—(x w1118) (n = 6 VNCs) and wor > nrx-IV RNAiBDSC32424 (n = 7 VNCs). Data statistics: Mann–Whitney U test. Results are presented as box and whisker plots, where whiskers mark the minimum and maximum, the box includes the 25th–75th percentile, and the line in the box is the median. Individual values are superimposed. (D) Representative confocal pictures of the thoracic VNC for nrx-IV knockdown by RNAi (wor > nrx-IV RNAi, VDRC line GD9039) in NSC lineages (driver line Nrv2::GFP, wor-GAL4; tub-GAL80ts). n = 9 VNCs. Larvae are dissected after 68 h at 29°C from ALH0. CG membrane is visualised by Nrv2::GFP (green) and NSCs are labelled with anti-Dpn (grey). (E) Representative confocal pictures of thoracic VNCs for nrx-IV knockdown by RNAi (BDSC line 324242) in all neurons (ElaV-GAL4 driver, n = 7 VNCs), in mature neurons (nSyb-GAL4 driver, n = 7 VNCs), and in the CG (cyp4g15-GAL4 driver, n = [file pbio.3002352.s006.tif]

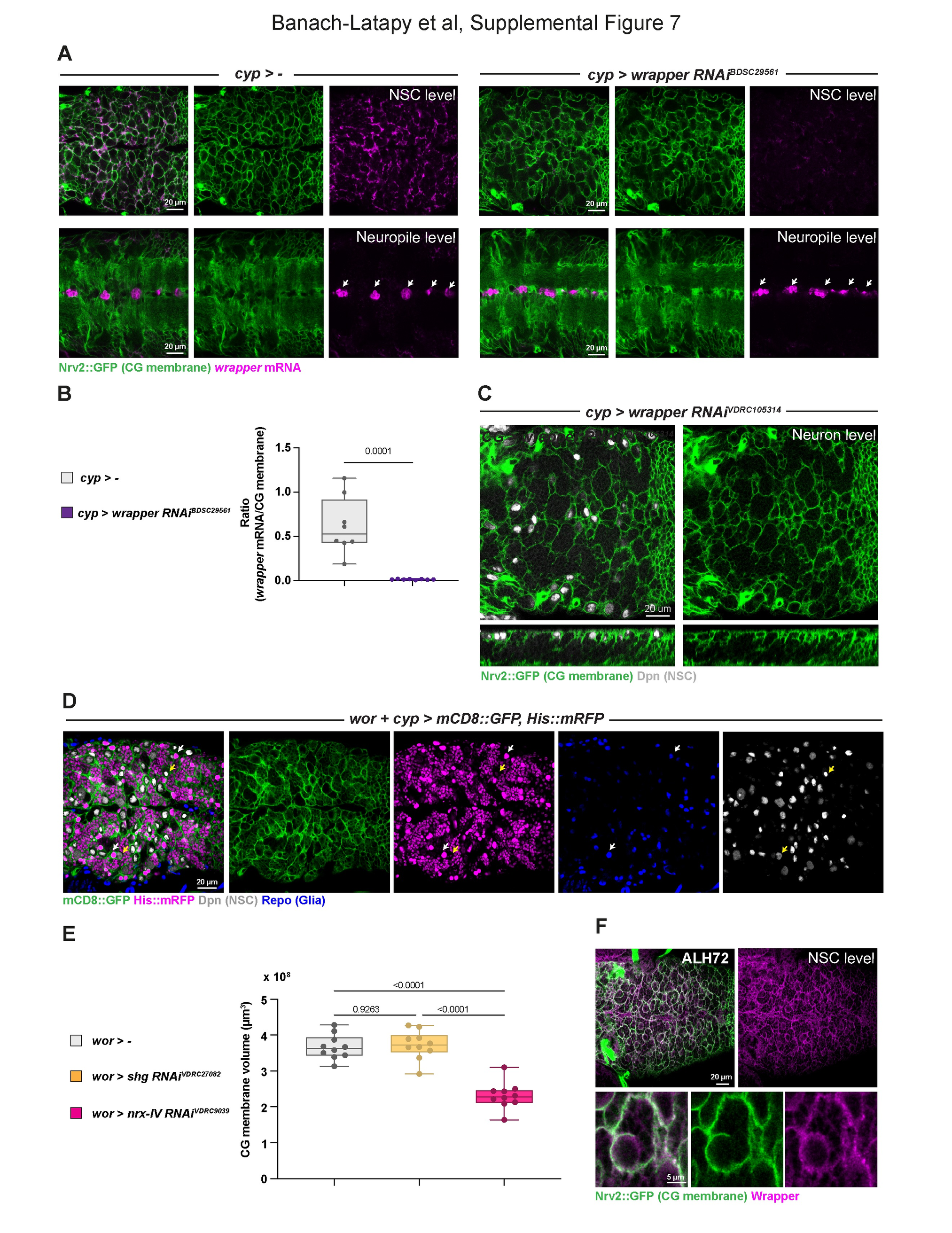

Supplement: S7 Fig — (A) Representative confocal pictures at the NSC and neuropile levels of the extent of loss of signal for wrapper (monitored through the detection by RNA FISH of wrapper mRNA, magenta) under wrapper knockdown (cyp > wrapper RNABDSC29561) in CG (driver line Nrv2::GFP, tub-GAL80ts; cyp4g15-GAL4) compared to control (cyp >—(x w1118)). Larvae are dissected after 68 h at 29°C from ALH0. White arrows indicate wrapper mRNA signal coming from the midline glia. (B) Quantification of the efficiency of wrapper knockdown in NSC lineages by RNAi from (A). wrapper levels are monitored through RNA FISH. See Methods for details of the quantification. cyp >—(x w1118) (n = 8 VNCs) and cyp > wrapper RNAiBDSC29561 (n = 8 VNCs). Data statistics: unpaired Student t test. Results are presented as box and whisker plots. (C) Representative confocal pictures of the thoracic VNC for wrapper knockdown by RNAi (cyp > wrapper RNAiVDRC105314) in the CG (driver line Nrv2::GFP, tub-GAL80ts; cyp4g15-GAL4). Larvae are dissected after 68 h at 29°C from ALH0. n = 8 VNCs. CG membrane is visualised by Nrv2::GFP (green), and NSCs are labelled with anti-Dpn (grey). (D) Representative confocal picture of the expression pattern of combined NSC lineages and CG drivers (wor + cyp) at the NSC level. UAS-mCD8::GFP and UAS-Histone2B::RFP were both driven under the control of Nrv2::GFP, worniu-GAL4; cyp4g15-GAL4, tub-GAL80ts. Larvae are dissected after 68 h at 29°C from ALH0. n = 6 VNCs. GAL4 expression is visualised by the membrane staining of mCD8::GFP (magenta) and nuclear staining of Histone2B::RFP (magenta). NSCs are labelled with anti-Dpn (grey) and glia with anti-Repo (blue). Expression in the CG is detectable by the stereotypic membrane pattern, and co-localisation between Histone2B::RFP and Repo (white arrows). Expression in NSC lineages is detectable by the co-localisation between Histone2B::RFP and Dpn (yellow arrows) and the accumulation of RFP+ progeny nuclei. (E) Quantification of the CG membrane volu [file pbio.3002352.s007.tif]

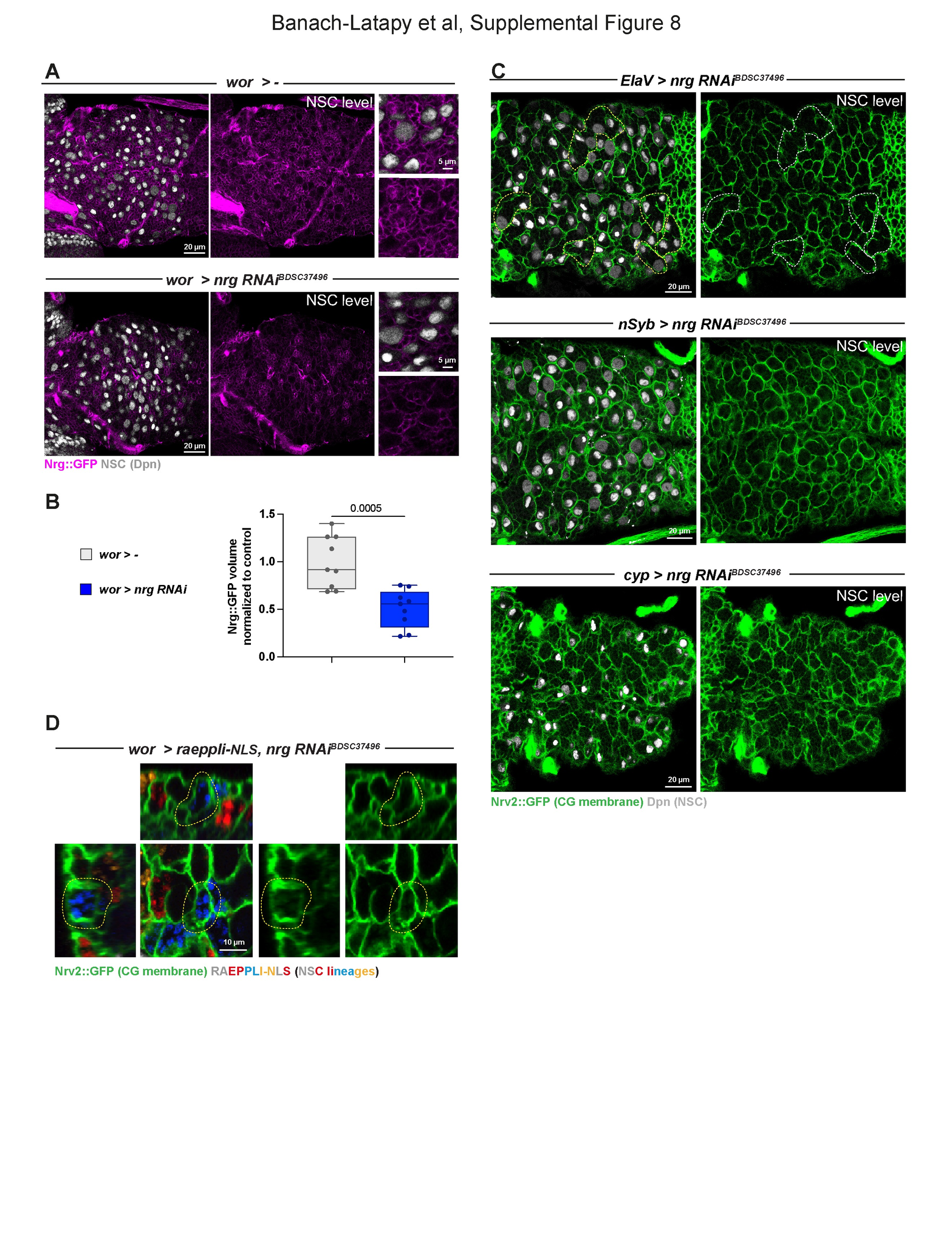

Supplement: S8 Fig — (A) Representative confocal pictures of the thoracic VNC for control (wor >—(x w1118)) and for nrg knockdown by RNAi (wor > nrg RNAiBDSC37496) in NSC lineages (driver line Nrg::GFP; cyp4g15-mtd::Tomato, wor-GAL4; tub-GAL80ts). Larvae are kept 24 h at 18°C, then dissected after 54 h at 29°C. Nrg levels are monitored through Nrg::GFP (magenta) and NSCs are labelled with Dpn (grey). (B) Quantification of the efficiency of nrg knockdown in NSC lineages by RNAi from (A). Nrg levels are monitored through Nrg::GFP. See Methods for details of the quantification. wor >—(x w1118) (n = 9 VNCs) and wor > nrg RNAiBDSC37496 (n = 9 VNCs). Data statistics: unpaired Student t test. Results are presented as box and whisker plots, where whiskers mark the minimum and maximum, the box includes the 25th–75th percentile, and the line in the box is the median. Individual values are superimposed. (C) Representative confocal pictures of thoracic VNCs for nrg knockdown (BDSC line 37496) by RNAi in all neurons (ElaV-GAL4 driver, n = 8 VNCs), in mature neurons (nSyb-GAL4 driver, n = 7 VNCs), and in the CG (cyp4g15-GAL4 driver, n = 9 VNCs). For ElaV and nSyb, larvae are kept 24 h at 18°C then dissected after 54 h at 29°C. For cyp, larvae are dissected after 68 h at 29°C from ALH0. CG membrane is visualised by Nrv2::GFP (green), and NSCs are labelled with anti-Dpn (grey). (D) Confocal close-up pictures of a condition in which nrg is knocked down by RNAi (BDSC line 37496) in NSC lineages marked with the multicolour lineage tracing Raeppli-NLS (blue, white, orange, and red; see Fig 8D and 8E). Raeppli-NLS is induced at ALH0 using hs-Flp, and RNAi after 24 h at 18°C. Larvae are dissected 54 h after RNAi induction. CG membrane was visualised with Nrv2::GFP (green). See S1 Table for detailed genetics, timing, and conditions of larval rearing. The dashed yellow lines indicate a chamber in which only secondary neurons (blue) are found. The data underlying this figure’s quantifications can be found in S1 [file pbio.3002352.s008.tif]

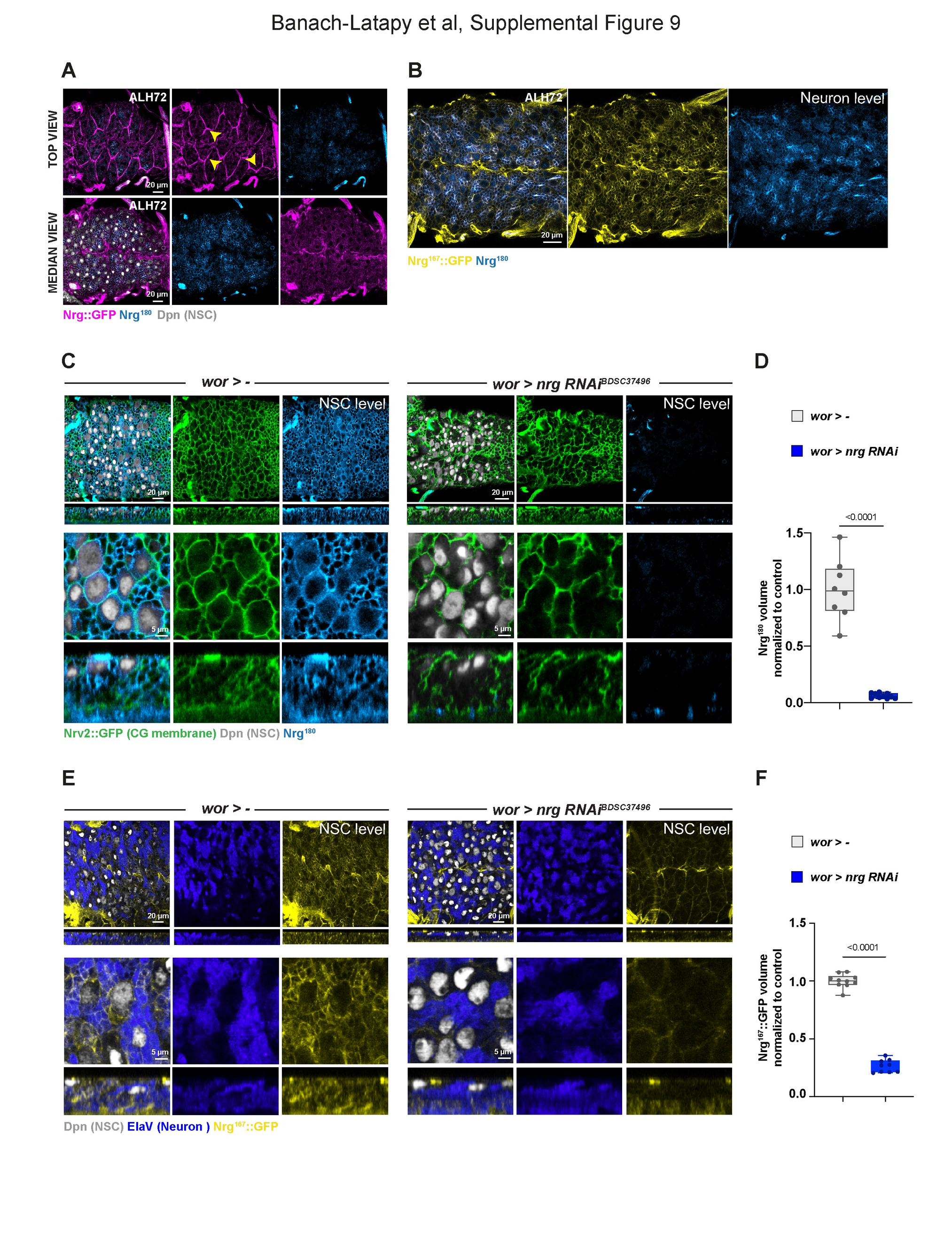

Supplement: S9 Fig — (A) Representative confocal pictures of the expression of all Nrg isoforms (Nrg::GFP, magenta) compared to the Nrg180 isoform (BP104, light blue) at ALH72 at 25°C. n = 8 VNCs. Upper panel, top view. Lower panel, median cut though the NSC population. Note the absence of signal at the septate junctions (yellow arrowheads on the top view, magenta signal) for Nrg180. (B) Representative confocal picture of the respective localisations of the Nrg167 and Nrg180 isoforms in a thoracic VNC, at ALH72 at 25°C. n = 6 VNCs. The Nrg167 isoform is visualised by a protein trap in the nrg gene leading to the preferential expression of this isoform (Nrg167::GFP, yellow). The Nrg180 isoform is detected with a specific antibody (BP104, light blue). (C) Representative confocal pictures and close-ups of thoracic VNCs for control (wor >—(x w1118)) and for nrg knockdown by RNAi (wor > nrg RNAi BDSC37496) in NSC lineages (driver line Nrv2::GFP, wor-GAL4; tub-GAL80ts). Larvae are kept 24 h at 18°C and then dissected after 54 h at 29°C. Levels of the Nrg180 isoform are monitored through staining with BP104 (light blue). CG membrane is visualised by Nrv2::GFP (green), and NSCs are labelled with anti-Dpn (grey). (D) Quantification of the efficiency of the knockdown of the Nrg180 isoform (BP104 antibody) in NSC lineages by RNAi (BDSC line 37496) from (C). See Methods for details of the quantification. wor >—(x w1118) (n = 8 VNCs) and wor > nrg RNAiBDSC37496 (n = 10 VNCs). Data statistics: unpaired Student t test. Results are presented as box and whisker plots. (E) Representative confocal pictures and close-ups of thoracic VNCs for control (wor >—(x w1118)) and for nrg knockdown by RNAi (wor > nrg RNAi BDSC37496) in NSC lineages (driver line Nrv2::GFP, wor-GAL4; tub-GAL80ts). Larvae are kept 24 h at 18°C and then dissected after 54 h at 29°C. Levels of the Nrg167 isoform are monitored through Nrg167::GFP (yellow). NSCs are labelled with anti-Dpn (grey) and neurons with anti-ElaV (blue). (F) Quant [file pbio.3002352.s009.tif]

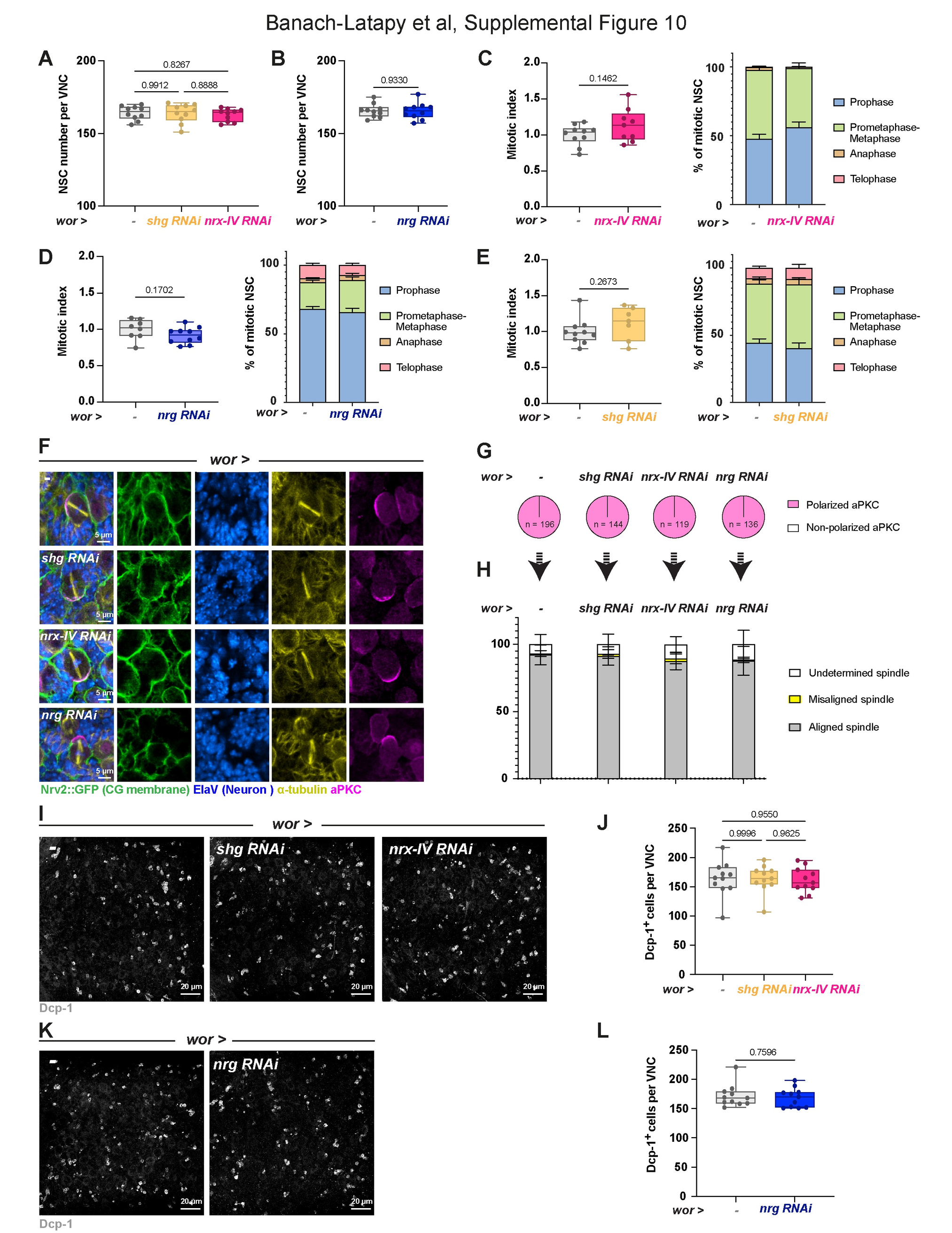

Supplement: S10 Fig — (A) Quantification of NSC number in the VNC for shg (line VDRC27082) knockdown and nrx-IV (line BDSC32424) knockdown in NSC lineages compared to control (driver line Nrv2::GFP, wor-GAL4; tub-GAL80ts). Larvae are dissected after 68 h at 29°C from ALH0. wor >—(x w1118) (n = 10 VNCs), wor > shg RNAIVDRC27082 (n = 10 VNCs), and wor > nrx-IV RNAIBDSC32424 (n = 10 VNCs). Data statistics: one-way ANOVA with Tukey’s multiple comparisons test. p = 0.8257 for the one-way ANOVA test on grouped dataset. P values from Tukey’s multiple comparisons test are displayed on the graph. Results are presented as box and whisker plots. (B) Quantification of NSC number in the VNC for nrg (line BDSC37496) knockdown in NSC lineages compared to control (driver line Nrv2::GFP, wor-GAL4; tub-GAL80ts). Larvae are dissected after 68 h at 29°C from ALH0. wor >—(x w1118) (n = 10 VNCs) and wor > nrg RNAIBDSC37496 (n = 10 VNCs). Data statistics: unpaired Student t test. Results are presented as box and whisker plots. (C) Mitotic index (left panel) and distribution of the mitotic phases (right panel) for control (wor >—(x w1118)) and nrx-IV knockdown (wor > nrx-IV RNAi, line BDSC32424) in NSC lineages (driver line Nrv2::GFP, wor-GAL4; tub-GAL80ts). Larvae are dissected after 68 h at 29°C from ALH0. wor >—(x w1118) (n = 10 VNCs) and wor > nrx-IV RNAIBDSC32424 (n = 9 VNCs). Data statistics: unpaired Student t test for mitotic index and two-way ANOVA with a Šidak’s multiple comparison test for mitotic phases. For the mitotic index, results are presented as box and whisker plots. For mitotic phases, stacked bars represent the respective percentage between aligned, misaligned, and undetermined spindles. Bars represent the SEM. There is no significant difference for any of the 4 phases. (D) Mitotic index (left panel) and distribution of the mitotic phases (right panel) for control (wor >—(x w1118)) and nrg knockdown (wor > nrg RNAi, line BDSC37496) in NSC lineages (driver line Nrv2::GFP, wor-GAL4; tub-GAL80 [file pbio.3002352.s010.tif]

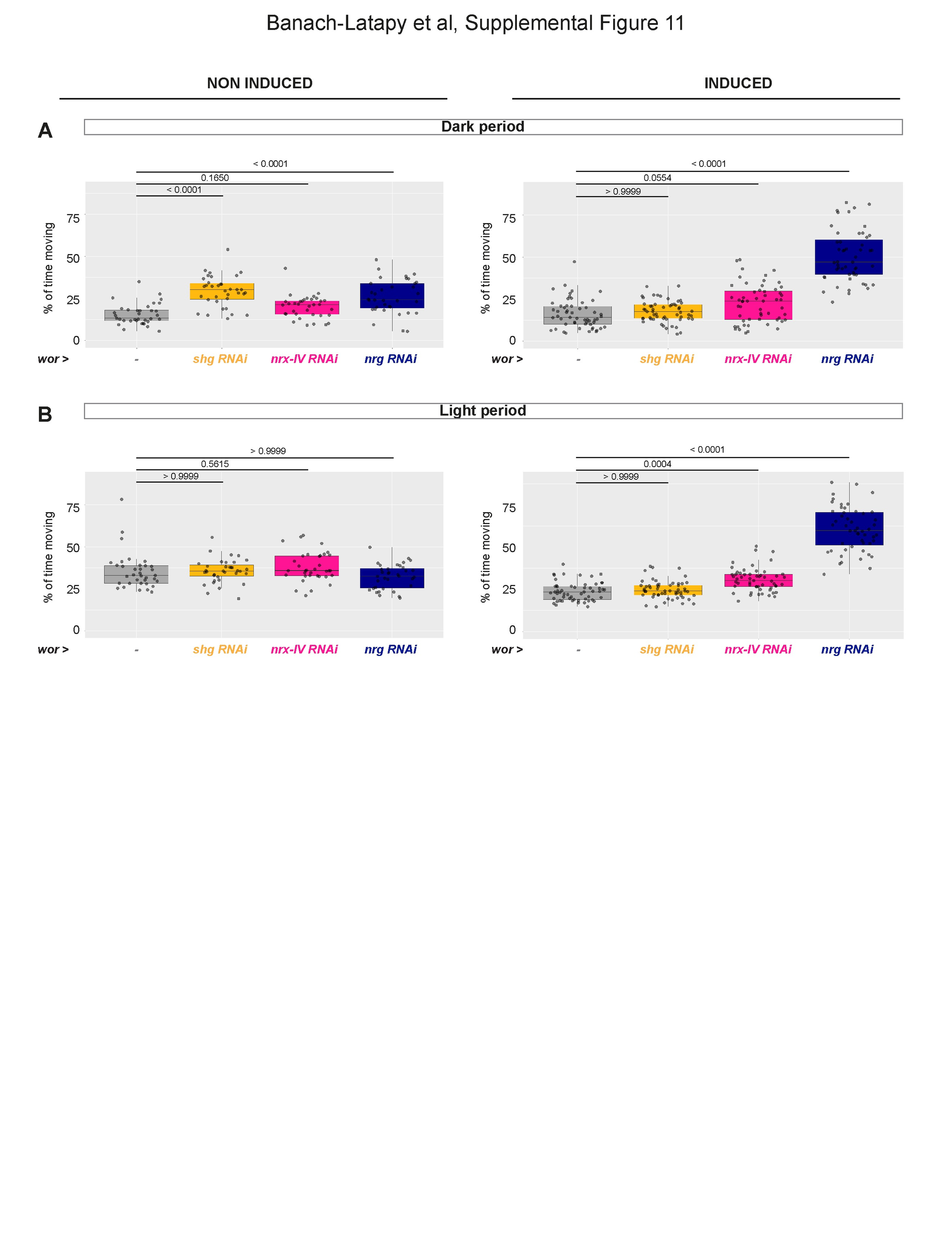

Supplement: S11 Fig — (A, B) For all metrics. Control (wor-GAL4, tub-Gal80ts x w1118), n = 35 non-induced adult males and n = 55 induced adult males. shg RNAi (wor-GAL4, tub-Gal80ts x shg RNAiVDRC27082), n = 35 non-induced adult males and n = 55 induced adult males. nrx-IV RNAi (wor-GAL4, tub-Gal80ts x nrx-IV RNAIBDSC32424), n = 35 non-induced adult males and n = 55 induced adult males. nrg RNAi (wor-GAL4, tub-Gal80ts x nrg RNAIBDSC37496), n = 35 non-induced adult males and n = 55 induced adult males. (A) Percentage of global time moving during the Dark period (% moving, ratio between total moving time and total time) in non-induced (flies always kept at 18°C before the recordings) and induced (flies shifted to 29°C from early larval stage to early pupal stage) conditions. Data statistics: Kruskal–Wallis H test with Dunn’s multiple comparisons test for both non-induced and induced conditions. p (non-induced) < 0.0001 and p (induced) < 0.0001 for the Kruskal–Wallis H test on grouped dataset. P values from Dunn’s multiple comparisons test are displayed on the graph. Results are presented as box and whisker plots. (B) Percentage of global time moving during the Light period (% moving, ratio between total moving time and total time) in non-induced (flies always kept at 18°C before the recordings) and induced (flies shifted to 29°C from early larval stage to early pupal stage) conditions. Data statistics: Kruskal–Wallis H test with Dunn’s multiple comparisons test for both non-induced and induced conditions. p (non-induced) = 0.0588 and p (induced) < 0.0001 for the Kruskal–Wallis H test on grouped dataset. P values from Dunn’s multiple comparisons test are displayed on the graph. Results are presented as box and whisker plots. For all box and whisker plots: whiskers mark the minimum and maximum, the box includes the 25th–75th percentile, and the line in the box is the median. Individual values are superimposed. The data underlying this figure’s quantifications can be found in S1 Data. Nrg, Ne [file pbio.3002352.s011.tif]

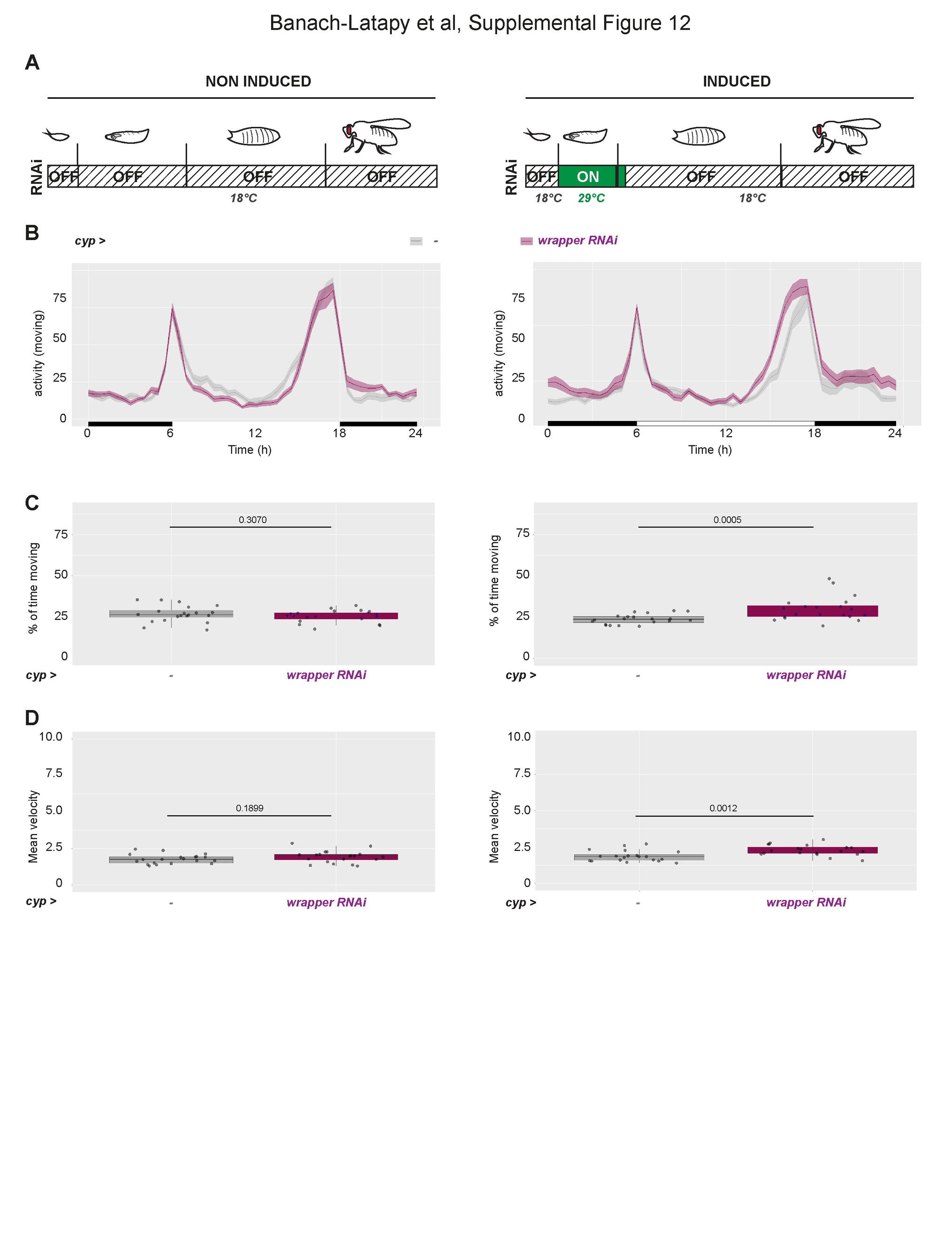

Supplement: S12 Fig — (A) Schematics of the rearing regimen for non-induced and induced conditions. In non-induced conditions, animals are constantly kept at 18°C before the recordings, a temperature allowing the repression of the GAL4/UAS system by the thermosensitive GAL80ts and thus blocking expression of the RNAi under the control of the CG driver (tub-Gal80ts; cyp4g15-GAL4). In induced conditions, the animals are transiently shifted to 29°C from the early larval stage to the early pupal stage, a temperature allowing the expression of GAL4/UAS system and thus of the RNAi. Animals were kept at 18°C both during the embryonic development and from the early pupal stage. Adult flies were assessed 7–10 days after eclosion. See S1 Table for detailed genetics, timing, and conditions of larval rearing. (B-D) Control, (tub-Gal80ts; cyp4g15-GAL4 x w1118), n = 20 non-induced adult males and n = 20 induced adult males. wrapper RNAi (tub-Gal80ts; cyp4g15-GAL4 x wrapper RNAiBDSC29561), n = 20 non-induced adult males and n = 20 induced adult males. (B) Plot representing the percentage of global time moving (measured as the fraction of time moving within 30-min intervals), in non-induced and induced conditions. (C) Fraction (%) of the time moving across LD cycles (% moving, ratio between total moving time and total time) in non-induced and induced conditions. Data statistics: unpaired Student t test for non-induced condition and Mann–Whitney U test for induced condition. Results are presented as box and whisker plots. (D) Mean velocity (in relative units) across LD cycles in non-induced and induced conditions. Data statistics: unpaired Student t test for both non-induced and induced conditions. Results are presented as box and whisker plots. For all box and whisker plots: whiskers mark the minimum and maximum, the box includes the 25th–75th percentile, and the line in the box is the median. Individual values are superimposed. The data underlying this figure’s quantifications can be found in S1 Data. [file pbio.3002352.s012.tif]

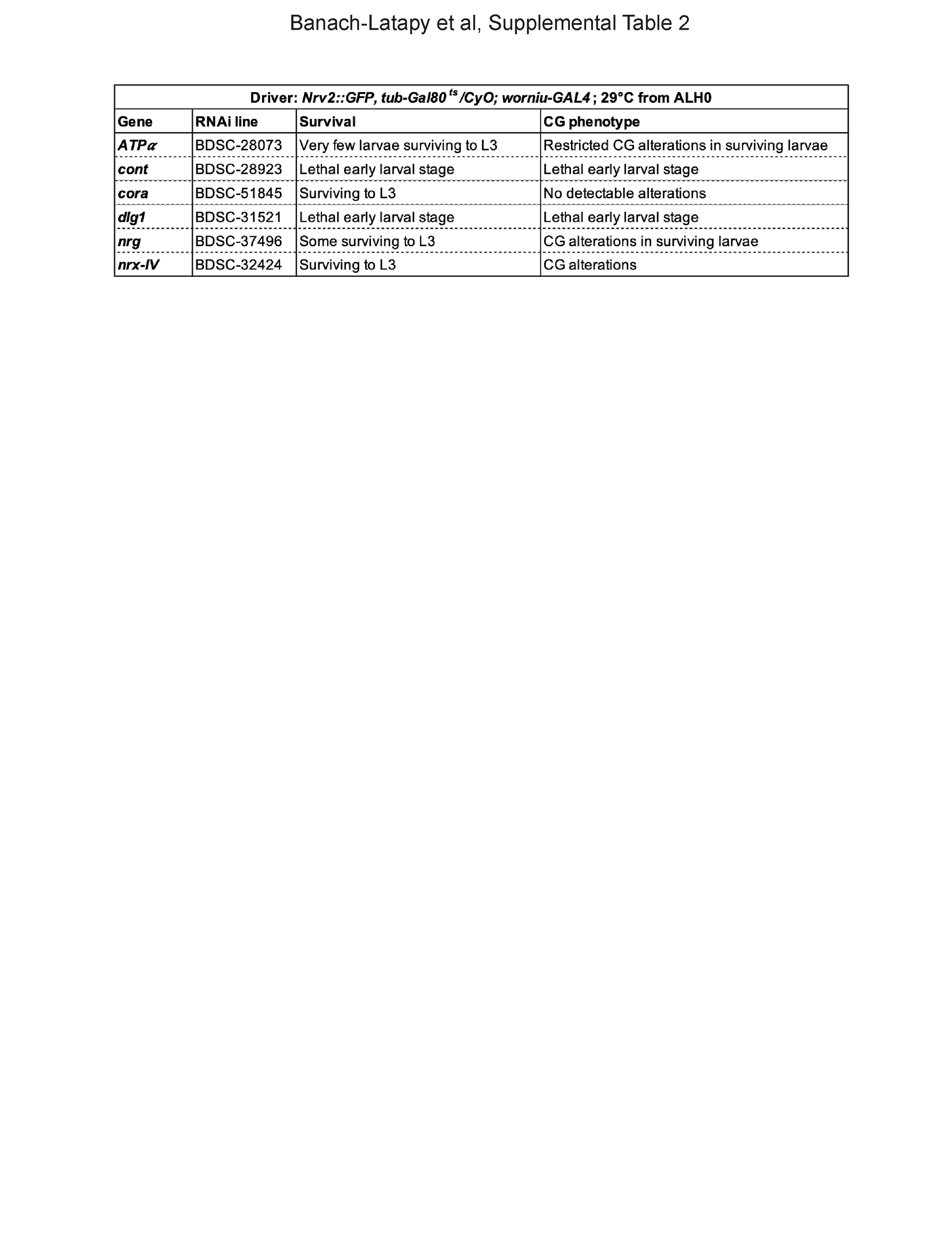

Supplement: S2 Table — RNAis against selected components of Drosophila occluding junctions were individually driven in the CG (driver line Nrv2::GFP, tub-GAL80ts; cyp4g15-GAL4), and larvae were dissected after 68 h at 29°C from ALH0. The CG membrane, labelled by Nrv2::GFP, was qualitatively assessed for alteration of normal overall morphology and individual encasing (n ≥ 7 VNCs). Change in survival was also recorded. (TIF) [file pbio.3002352.s014.tif]
