# Supplementary material for: Differential adhesion during development establishes individual neural stem cell niches and shapes adult behaviour in Drosophila
Source: PLoS Biol. 2023 Nov 9;21(11):e3002352. doi: 10.1371/journal.pbio.3002352 (PMC10635556; doi:10.1371/journal.pbio.3002352)
Supplement: S1 Table — (PDF) [file pbio.3002352.s013.pdf]

Supplemental Table 1. Genotypes, crosses and regimen of fly culture

Figures	Genotypes and crosses	Regimen
1C, F<br>Supp. Fig. 4B<br>Supp. Fig. 7D	*Nrv2::GFP/CyO*	ALH72 at 25°C
2A-B<br>Supp. Fig. 4A	*Nrv2::GFP, worniu-GAL4/CyO; tub-GAL80^{ts} x UAS -pros RNAi*<br>*x UAS-brat RNAi*<br>*x w^{1118}*	For *pros* tumours:<br>- 18°C until larval hatching (ALH0)<br>- 48 h at 18°C<br>- 48-52 h at 29°C<br><br>For *brat* tumours:<br>- 18°C until larval hatching (ALH0)<br>- 72 h at 29°C
2C-D<br>Supp. Fig. 2C-F	*yw, hs-FLP ; Nrv2::GFP, wor-GAL4/CyO*<br><br>*x UAS-Raeppli-NLS 53D; UAS-pros RNAi, tubGAL80^{ts}*<br>*x UAS-Raeppli-NLS 53D; UAS-brat RNAi, tubGAL80^{ts}*<br>*x UAS-Raeppli-NLS 53D; tubGAL80^{ts}*	For *pros* tumours:<br>- 18°C until larval hatching (ALH0)<br>- 24 h at 18°C<br>- 2 h heatshock at 37°C<br>- 48-52 h at 29°C<br><br>For *brat* tumours:<br>- 18°C until larval hatching (ALH0)<br>- 48 h at 18°C<br>- 2 h heatshock at 37°C / no HS<br>- 48-52 h at 29°C
2E	*grh-FLP ; CoinFLP GAL4::LexA*<br>*x Nrv2::GFP, UAS-mCD8::RFP/CyO*<br>*x Nrv2::GFP/CyO, UAS-mCD8::RFP; UAS-brat RNAi*	ALH72-96 at 25°C
3B-C<br>Supp. 3A-B<br>5B-C<br>Supp. Fig. 5A-C	*tub-QS; Nrv2::GFP, wor-GAL4/CyO*<br>*x yw, hs-FLP; UAS-Raeppli-NLS 53D*<br>*x yw, hs-FLP; UAS-Raeppli-NLS 53D, QUAS-PTEN*<br>*x yw, hs-FLP; UAS-Raeppli-NLS 53D, QUAS-PTEN; UAS-shg RNAi^{VDRC27082}*	- Heatshock 2 h 37°C at ALH0<br>- 72 h at 29°C on plates with 20 mg/ml quinic acid (T1) followed by 28h at 29°C on plates without quinic acid (T2)
3E-F	*cyp4g15-FLP ; CoinFLP GAL4::LexA ; UAS-mCD8::RFP*<br>*x Nrv2::GFP/CyO*<br>*x Nrv2::GFP/CyO; UAS-PTEN*	ALH48-72 at 25°C
4A	*cyp4g15-mtd::Tomato x shg::GFP*	ALH0, ALH24, ALH48, ALH72 at 25°C
4C	*Nrv2::GFP, worniu-GAL4/CyO; tub-GAL80^{ts} x UAS-shg RNAi^{VDRC27082}*	- 18°C until larval hatching (ALH0)<br>- 68 h at 29°C
4E-F	*Nrv2::GFP; wor-GAL4/CyO;*<br>*x yw, hs-FLP; UAS-Raeppli-nls 53D; UAS-shg RNAi^{VDRC27082}*<br>*x yw, hs-FLP*	- 18°C until larval hatching (ALH0)<br>- Heatshock 2 h 37°C at ALH0<br>- 72 h at 29°C
5D	*Nrv2::GFP, worniu-GAL4/CyO; tub-GAL80^{ts} x UAS-shg*	- 18°C until larval hatching (ALH0)<br>- 68 h at 29°C
6A	*cyp4g15-mtd::Tomato x Nrx-IV::GFP*	ALH0, ALH24, ALH48, ALH72 at 25°C
6B	*Nrv2::GFP, worniu-GAL4/CyO; tub-GAL80^{ts} x UAS-nrx-IV RNAi^{BDSC32424}*	- 18°C until larval hatching (ALH0)<br>- 68 h at 29°C
6D-E	*Nrv2::GFP; wor-GAL4/CyO; UAS-Raeppli-nls 89A*<br>*x yw, hs-FLP; UAS-Nrx-IV RNAi^{BDSC32424}*<br>*x yw, hs-FLP*	- 18°C until larval hatching (ALH0)<br>- Heatshock 2 h 37°C at ALH0<br>- 72 h at 29°C

7A	*Nrv2::GFP/CyO*	ALH0, ALH24, ALH48, ALH72 at 25°C
7B	*Nrv2::GFP, tub-GAL80^{ts}/CyO; cyp4g15-GAL4*<br>*X UAS-wrapper RNAi^{BDSC29561}*	- 18°C until larval hatching (ALH0)<br>- 68 h at 29°C
7C-D	*Dpn-FRT-STOP-FRT-LexA; cyp4g15-GAL4, LexAOp-Raeppli-NLS 89A*<br>*x hs-FLP; Nrv2::GFP, tub-GAL80^{ts}; UAS-wrapper RNAi^{BDSC29561}*<br>*x hs-FLP; Nrv2::GFP, tub-GAL80^{ts};*	- 18°C until larval hatching (ALH0)<br>- Heatshock 2 h 37°C at ALH0<br>- 72 h at 29°C
7E-F	*Nrv2::GFP, worniu-GAL4/CyO;cyp4g15-GAL4, tub-GAL80^{ts}*<br>*x UAS-RFP RNAi; UAS-nrx-IV RNAi^{BDSC32424}*<br>*x UAS-wrapper RNAi^{VDRC105314}; UAS-mCherry RNAi*<br>*x UAS-wrapper RNAi^{VDRC105314}; UAS-nrx-IV RNAi^{BDSC32424}*	- 18°C until larval hatching (ALH0)<br>- 68 h at 29°C
7G	*Nrv2::GFP, worniu-GAL4/CyO; tub-GAL80^{ts} x UAS-wrapper*<br>*x w^{1118}*	- 18°C until larval hatching (ALH0)<br>- 68 h at 29°C
8A	*Nrg::GFP*	ALH0, ALH24, ALH48, ALH72 at 25°C
8B	*Nrv2::GFP, worniu-GAL4/CyO; tub-GAL80^{ts} x UAS-nrg RNAi^{BDSC37496}*	- 18°C until larval hatching (ALH0)<br>- 24 h at 18°C<br>- 54 h at 29°C
8D-E<br>Supp. Fig. 8D	*yw, hs-FLP; Nrv2::GFP; wor-GAL4/CyO; tub-Gal80^{ts}*<br>*x UAS-nrg RNAi ; UAS-Raeppli-NLS 89A*<br>*x UAS-Raeppli-NLS 89A*	- 18°C until larval hatching (ALH0)<br>- Heatshock 2 h 37°C at ALH0<br>- 24 h at 18°C<br>- 60 h at 29°C
9B<br>Supp. Fig. 9A	*Nrg::GFP*	ALH72 at 25°C
9C<br>Supp. Fig. 9B	*Nrg^{167}::GFP*	ALH72 at 25°C
9D-F	*Nrv2::GFP, tub-GAL80^{ts}/CyO; cyp4g15-GAL4*<br>*x UAS-Nrg^{180}*<br>*x UAS-Nrg^{167}*<br>*x UAS-Nrg^{GPI}*<br>*x w^{1118}*	- 18°C until larval hatching (ALH0)<br>- 68 h at 29°C
9G-I	*Nrv2::GFP, worniu-GAL4/CyO; tub-GAL80^{ts}*<br>*x UAS-Nrg^{180}*<br>*x UAS-Nrg^{167}*<br>*x UAS-Nrg^{GPI}*<br>*x w^{1118}*	- 18°C until larval hatching (ALH0)<br>- 68 h at 29°C
10B,C,E	*yw, hs-FLP; Nrv2::GFP; wor-GAL4/CyO; tub-Gal80^{ts}*<br>*x UAS-Raeppli-CAAX 42D*<br>*x UAS-Raeppli-CAAX 42D ; UAS-shg RNAi^{VDRC27082}*<br>*x UAS-Raeppli-CAAX 42D ; UAS-Nrx-IV RNAi^{BDSC32424}*	- 18°C until larval hatching (ALH0)<br>- Heatshock 2 h 37°C at ALH0<br>- 72 h at 29°C
10C,F	*yw, hs-FLP ; Nrv2::GFP, wor-GAL4/CyO; tub-GAL80^{ts}*<br>*x UAS-Raeppli-CAAX 99E*<br>*x UAS-nrg RNAi^{BDSC37496}, UAS-Raeppli-CAAX 99E*	- 18°C until larval hatching (ALH0)<br>- Heatshock 2 h 37°C at ALH0<br>- 24 h at 18°C<br>- 60 h at 29°C
10H, J	*Nrv2::GFP, worniu-GAL4/CyO; tub-GAL80^{ts}*	- 18°C until larval hatching (ALH0)

Supp. Fig. 7E<br>Supp. Fig. 10A, E	*x UAS-shg RNAi*^{VDRC27082}<br>*x UAS-nrx-IV RNAi*^{BDSC32424}<br>*x w*^{1118}	- 68 h at 29°C
10I,L	*Nrv2::GFP, worniu-GAL4/CyO; tub-GAL80*^{ts}<br>*x UAS-nrg RNAi*^{BDSC37496}<br>*x w*^{1118}	- 18°C until larval hatching (ALH0)<br>- 24 h at 18°C<br>- 54 h at 29°C
11<br>Supp. Fig. 11	*worniu-GAL4, tub-GAL80*^{ts}*/CyO*<br>*x UAS-shg RNAi*^{VDRC27082}<br>*x UAS-nrx-IV RNAi*^{BDSC32424}<br>*x UAS-nrg RNAi*^{BDSC37496}<br>*x w*^{1118}	Non-induced conditions:<br>- 18°C until observation<br>- locomotor test after 7-10 of adulthood, performed at 22°C<br><br>Induced conditions:<br>- 18°C until larval hatching (ALH0)<br>- 0 to 24 h at 18°C<br>- 29°C until early pupae (wandering L3 +0-24 h)<br>- locomotor test after 7-10 of adulthood, performed at 22°C
Supp. Fig. 1A	*yw, hs-FLP ; Nrv2::GFP, wor-GAL4/CyO; tub-GAL80*^{ts}<br>*x UAS-Raeppli-CAAX 99E*	- 18°C until larval hatching (ALH0)<br>- 68 h at 29°C
Supp. Fig. 1B	*worniu-GAL4, UAS-myr::mCherry/CyO, GFP*	ALH72 at 25°C
Supp. Fig. 1C	*worniu-GAL4, UAS-myr::mCherry/CyO, GFP; tub-GAL80*^{ts}<br>*x UAS-mCherry RNAi*<br>*x UAS-GFP RNAi*	- 18°C until larval hatching (ALH0)<br>- 68 h at 29°C
Supp. Fig. 2A	*Nrv2::GFP, worniu-GAL4/CyO; tub-GAL80*^{ts}<br>*x UAS-Dpn*<br>*x UAS-myr-aPKC*<br>*x w*^{1118}	For *aPKc* tumours:<br>- 18°C until larval hatching (ALH0)<br>- 48 h at 18°C<br>- 48-52 h at 29°C<br><br>For *dpn* tumours:<br>- 18°C until larval hatching (ALH0)<br>- 72 h at 29°C
Supp. Fig. 2B	*Nrv2::GFP/CyO*	ALH0, ALH16, ALH24, ALH30, ALH48, ALH72, ALH96 at 25°C
Supp. Fig. 3B-C	*cyp4g15-FLP ; CoinFLP GAL4::LexA ; UAS-mCD8::RFP*<br>*x Nrv2::GFP, tub-GAL80*^{ts} */CyO*<br>*x Nrv2::GFP, tub-GAL80*^{ts}*/CyO; UAS-reaper*	- 18°C until larval hatching (ALH0)<br>- 96 h at 18°C<br>- 48 h at 29°C
Supp. Fig. 4C	*TUG13 MARCM line (y,w, hs-FLP ; FRTG13, tubP-GAL80[LL2]/ (CyO, act-GFP[JMR1]) ; tubP-GAL4[LL7], UAS-mCD8-GFP[LL6]/TM6B)*<br>*x shg64R, FRT42B; cyp4g15-mtd::Tomato*<br>*x FRT42B; cyp4g15-mtd::Tomato*	- 25°C throughout development<br>- 37°C heatshock:<br>14-18 h after egg laying for 2 h<br>Or<br>ALH48 for 30 min<br>- dissection at ALH72
Supp. Fig. 4D-E	*shg::GFP x w*^{1118}<br><br>*shg::GFP; worniu-GAL4*<br>*x UAS-shg RNAi*^{VDRC27082}<br>*x UAS-shg RNAi*^{VDRC103962}<br><br>*shg::GFP; cyp4g15-GAL4*<br>*x UAS-shg RNAi*^{VDRC27082}	- 18°C until larval hatching (ALH0)<br>- 68 h at 29°C

Supp. Fig. 4F	*Nrv2::GFP, tub-GAL80^{ts}/CyO; cyp4g15-GAL4*<br>*X shg RNAi^{VDRC103962}*	18°C until larval hatching (ALH0)
Supp. Fig. 6A	*Nrv2::GFP*<br>*ATP::GFP*<br>*dlg1::GFP*<br>*Nrx-IV::GFP*<br>*Nrg::GFP*	ALH72 at 25°C
Supp. Fig. 6B-C<br>Supp. Fig. 10C	*Nrv2::GFP, worniu-GAL4/CyO; tub-GAL80^{ts} x UAS-nrx-IV RNAi^{BDSC32424}*<br>*x w^{1118}*	- 18°C until larval hatching (ALH0)<br>- 68 h at 29°C
Supp. Fig. 6D	*Nrv2::GFP, worniu-GAL4/CyO; tub-GAL80^{ts} x UAS-nrx-IV RNAi^{VDRC9039}*	- 18°C until larval hatching (ALH0)<br>- 68 h at 29°C
Supp. Fig. 6E	*UAS-nrx-IV RNAi^{BDSC32424}*<br>*x ElaV-GAL4; Nrv2 ::GFP, tub-GAL80^{ts}/CyO;*<br>*x Nrv2::GFP, nSyb-GAL4/CyO; tub-GAL80^{ts}*<br>*x Nrv2::GFP, tub-GAL80^{ts}/CyO; cyp4g15-GAL4*	- 18°C until larval hatching (ALH0)<br>- 68 h at 29°C
Supp. Fig. 7A-B	*Nrv2::GFP, tub-GAL80^{ts}/CyO; cyp4g15-GAL4*<br>*x UAS-wrapper RNAi^{BDSC29561}*<br>*x w^{1118}*	- 18°C until larval hatching (ALH0)<br>- 68 h at 29°C
Supp. Fig. 7C	*Nrv2::GFP, tub-GAL80^{ts}/CyO; cyp4g15-GAL4*<br>*x UAS-wrapper RNAi^{VDRC105314}*	- 18°C until larval hatching (ALH0)<br>- 68 h at 29°C
Supp. Fig. 7F	*worniu-GAL4/CyO;cyp4g15-GAL4*<br>*x UAS-mCD8::GFP, UAS-Histone2b::RFP*	ALH72 at 25°C
Supp. Fig. 8A-B	*Nrg::GFP, worniu-GAL4/CyO; tub-GAL80^{ts} x UAS-nrg RNAi^{BDSC37496}*<br>*x w^{1118}*	- 18°C until larval hatching (ALH0)<br>- 24 h at 18°C<br>- 54 h at 29°C
Supp. Fig. 8C	*UAS- nrg RNAi^{BDSC37496}*<br>*x ElaV-GAL4; Nrv2 ::GFP, tub-GAL80^{ts}/CyO;*<br>*x Nrv2::GFP, nSyb-GAL4/CyO; tub-GAL80^{ts}*<br>*x Nrv2::GFP, tub-GAL80^{ts}/CyO; cyp4g15-GAL4*	For *ElaV-GAL4* and *nSyb-GAL4*:<br>- 18°C until larval hatching (ALH0)<br>- 24 h at 18°C<br>- 54 h at 29°C<br><br>For *cyp4g15-GAL4*:<br>- 18°C until larval hatching (ALH0)<br>- 68 h at 29°C
Supp. Fig. 9C-D<br>Supp. Fig. 10B, D, F-G, K-L	*Nrv2::GFP, worniu-GAL4/CyO; tub-GAL80^{ts} x UAS-nrg RNAi^{BDSC37496}*<br>*x w^{1118}*	- 18°C until larval hatching (ALH0)<br>- 24 h at 18°C<br>- 54 h at 29°C
Supp. Fig. 9E-F	*Nrg^{167}::GFP; worniu-GAL4, tub-Gal80^{ts}*<br>*x UAS- nrg RNAi^{BDSC37496}*<br>*x w^{1118}*	- 18°C until larval hatching (ALH0)<br>- 24 h at 18°C<br>- 54 h at 29°C

Supp. Fig. 10F-J	*Nrv2::GFP, worniu-GAL4/CyO; tub-GAL80^{ts}*<br>*x UAS-shg RNAi^{VDRC27082}*<br>*x UAS-nrx-IV RNAi^{VDRC9039}*<br>*x w^{1118}*	- 18°C until larval hatching (ALH0)<br>- 68 h at 29°C
Supp. Fig. 12	*tub-GAL80^{ts}; cyp4g15-GAL4*<br>*x UAS-wrapper RNAi^{VDRC105314}*<br>*x w^{1118}*	Non-induced conditions:<br>- 18°C until observation<br>- locomotor test after 7-10 of adulthood, performed at 22°C<br><br>Induced conditions:<br>- 18°C until larval hatching (ALH0)<br>- 0 to 24 h at 18°C<br>- 29°C until early pupae (wandering L3 +0-24 h)<br>- locomotor test after 7-10 of adulthood, performed at 22°C
