# Supplementary material for: Differential adhesion during development establishes individual neural stem cell niches and shapes adult behaviour in Drosophila
Source: PLoS Biol. 2023 Nov 9;21(11):e3002352. doi: 10.1371/journal.pbio.3002352 (PMC10635556; doi:10.1371/journal.pbio.3002352)
Supplement: Attachment — Submitted filename: ResponseReviewers_BanachLatapy.pdf [file pbio.3002352.s016.pdf]

Response to reviewers' comments of manuscript PBIOLOGY-D-22-02564R1

We first would like to thank the reviewers for their critical reading of our findings, and their suggestions to improve our manuscript. We have now addressed the different points which were raised either by providing new experimental data or by clarifying the text and figures.

New or updated figures are:
• Fig. 1F-G" and Supp. Fig. 1B-C
• Supp. Fig. 2C-D
• Fig. 3D,F and Supp Fig. 3C
• Fig. 4C-D and Supp. Fig. 4E
• Fig. 6B-C and Supp. FIG. 6C, D and E (*nSyb-GAL4*)
• Fig. 7 and Supp. Fig. 7A-E
• Fig. 8B-C and Supp. Fig. 8B and 8C (*nSyb-GAL4*)
• Fig 9E-I and Fig. 9D-F
• Fig. 10 G-K and Supp. Fig 10A-B and F-L
• Fig. 12 (model)

The major additions or changes in the text are written in blue.

Reviewer #1: Proper interactions between somatic stem cells and their surrounding niche microenvironments play a key role in ensuring normal tissue development. Despite their critical roles in promoting stem cell functions, the mechanisms that coordinate the formation of precise niche architecture relative to the timing of stem cell proliferation during organogenesis remain poorly understood. In the developing Drosophila larval brain, cortex glia directly contacts NSCs and their progeny, and form discrete glial compartments that encapsulate individual NSC lineages. Cortex glia functions as niches that promote NSC proliferation, resistance to stress and survival. Cortex glial chambers ensure that clonally derived stem cell progeny acquire temporally specific neuronal identities and contribute to proper formation of neuronal circuits during post-embryonic brain neurogenesis. Previous studies have suggested that post-embryonic nutrition intake initiates the encapsulation of NSCs by cortex glia and NSC exit from quiescence. By using this in vivo paradigm, the study by Banach-Latapy et al. investigates the mechanisms that coordinate establishment of cortex glial niches relative to stem cell proliferation. The authors identified two adhesion molecules, Neurexin-IV (Nrx-IV) and Neuroglian (Nrg), that are required for proper encasing of NSC lineages by cortex glia. Loss of nrx-IV or nrg function perturbs the projection of axonal bundles during post-embryonic neurogenesis and leads to defective locomotor functions in adult flies.

The reviewers appreciate elegant experimental approaches taken by the authors to investigate NSC-glia interactions under the physiological context and the impact on brain functions. However, the reviewers are uncertain whether defects in neurogenesis and cell death rather than cortex glial chamber formation lead to defective axonal projection and observed behavioral defects in mutant flies. Reviewers' concerns stem from phenotypic assessment and data interpretations associated with Figures 4-5. For example, knocking down nrx-IV function in NSCs appears to reduce NSC numbers (Figure 4C) comparing to control shown in Figure 3C. The authors should compare the total number of NSCs in the nrx-IV knock-down VNC to the age-matched control VNC. If NSC numbers are decreased following nrx-IV knock-down in the VNC, the phenotypes associated with axon projection shown in Figure 6 are due to defects in neurogenesis or cell death rather than formation of glial chambers. Similarly, knocking down nrg function in NSCs appears to increase NSC numbers (Figure 4F) comparing to control shown in Figure 3C. The authors should compare the total number of NSCs in the nrg knock-down VNC to the age-matched control VNC, and determine whether GMCs acquire NSC identities at the expense of neuron generation. Similar caveats can be seen in Figure 5G-I. If precise quantification of total NSC numbers is impossible, the authors should perform mosaic clonal analyses in these experiments to determine whether manipulation of nrx-IV or nrg functions leads to cell fate misspecification or cell death. Alternatively, the authors can repeat these manipulations by using a Gal4 driver that is active in a small subset of NSCs in the VNC.
If manipulation of nrx-IV or nrg function in NSCs or cortex glia indeed perturbs differentiation of NSC progeny and results in ectopic NSC formation, the authors should perform additional experiments to characterize these interesting phenotypes and discuss how these adhesion molecules promote proper cell fate specification.

We agree with Reviewer 1 that defects in NSC features could impact overall neuronal connections and function further, and as such should be taken in consideration. We did look at it at the time but failed to report it, so we thank the Reviewers for this opportunity.
1. The apparent difference in NSC numbers in previous Figure 4 and 5 compared to control stems from a difference in the plane of view. We choose different plans to highlight the difference in CG phenotype between these two conditions, something we tried to explain using the white dashed line in the orthogonal view, and in the figure legends. We realise we did not convey this idea clearly. We have now created a novel Figure 1 in which we explain and represent the different plans used (NSC, Neuron). We also further use these nomenclature and representations to explain the different phenotypes.
2. We had quantified NSC numbers in the different conditions to calculate the percentage of proliferating cells (previous Supp Fig. 6A-C) and found no significant change. We are sorry we did not represent these data at the time. We have now added the corresponding graphs and statistics as Supp. Fig. 10A-B.

3. To complete our results on NSC proliferation, we have added data on aPKC polarization, a marker of asymmetric division, by quantifying it orientation with respect to the mitotic spindle and the metaphasic plate. These data are represented in Supp. Fig. 10F-H.
4. Finally, we have performed a Dcp-1 (*Drosophila* cleaved caspase 1) staining as a marker for apoptotic cells and found no difference in the total number of Dcp-1+ cells per VNC in the different conditions. These data are represented in Supp. Fig. 10I-L.
We believe these data support that NSCs do not die, do not display measurable defects in proliferation and do not undergo altered differentiation.

Minor points
1. The authors claimed that tumor NSCs derived from single NSC are encased by a single cortex glial chamber in Figure 1F & 1G. If this is the case, there should only be 8 cortex glial chambers in brains where brat function was knocked down. Please provide quantification of cortex glial chambers that encapsulate type II NSCs in brat-mutant brains. If there are more than 8 cortex glial chambers, please discuss the discrepancy in the discussion.

We have now added these data as Supp. Fig. 1C, and the quantification is explained in the Methods lines 239-246. Quantifying the number of CG chamber is challenging in *brat* tumours due to their size and geometry, which makes following the continuity of their outline complicated. We thus only quantified the tissues in which we were confident in x,y, z of what we were seeing.
We could not always detect 8 chambers both for *brat* and control, but rather less. Some chambers become more difficult to see depending on variation in sample mounting and staining or Raeppli induction. We do not find more than 8 chambers in any of the 2 conditions.
Of note, we noticed that some chambers (few in control, more in *brat* due to the overproliferation) contained several colours of Raeppli-NLS. We believe it is due to the later uncontrolled activation of Raeppli-NLS in INPs or tumour NSCs in which the original heatshock-controlled recombination failed. This would result in differently-marked INP/tumour NSC clones, yet from the same original NSC mother cell. We have also added these data as Supp. Fig. 1D and commented in the text (Results lines 37-43).

2. Please quantify the number of NSCs per glial chamber in Figure 3D.

It was originally shown in Supp. Figure 3G, we are sorry its location was not obvious. After reorganizing the figures, this quantification is now included as Fig. 4F.

3. Overall, we would like to the authors to improve their figure captions to help readers better appreciate the data in all figures instead of methodology.

We understand from all reviewers that they found our captions too restricted. We originally did so in purpose to avoid redundancy (*e.g.*, repeating the features of the Whisker-and-box plots) and ease the reading for non-Drosophila readers (*e.g.*, keep complex genetics for the Methods). We have now brought back some of these elements from the Methods to the legends, and added a double nomenclature for the stainings on the figure itself to help associating molecular markers with cellular features (*e.g.*, Nvr2::GFP/CG membrane). We still think some of the genetics are too heavy for the legends, and we have decided to gather all the genotypes and conditions in Supp. Table 1, what we hope should make them accessible easily in one place.

4. Images that are shown in the X-Y axis and the Z-axis in Figures 1 & 2 are very difficult to interpret. Please highlight a single representative glial chamber in these panels to illustrate the interpretation.

We have now done so.

Reviewer #2: The manuscript "Differential adhesion during development establishes individual neural stem cell niches and controls adult behaviour" provides an interesting analysis of the role of differential adhesion in separating neural stem cell lineages during CNS development in Drosophila. This paper provides clear evidence that two membrane proteins (Nrx-IV and Neuroglian - with functions known to provide adhesion at septate junctions) also play a role in maintaining the segregation of lineages. Loss of either leads to the mixing or grouping of several lineages leading to disruption in axon projections and locomotor hyperactivity in adults.

Overall, this was a well written and elegant analysis of the role that cortex glial play in NSC lineage segregation. The figures were compelling and clearly presented. In particular the authors made use of several clever approaches and made excellent use of multi-colour clonal analysis (Raeplli system) to follow the fate of NSC and their progeny. A major addition was the use of cartoons throughout to illustrate the processes being analyzed. Some additional clarification and explanations for experiments and genetic manipulations would strengthen the manuscript and some sections the conclusions should be more qualified given the data presented. Specific comments about the data are outlined below tied to their appropriate figures.

Figure 1 in particular did an excellent job of introducing the cell types and their interactions over developmental time. This figure could have been improved by instead of using AL > AL-CG using more words in the figure to describe this as it was not intuitive. For Figure 1F what was the control cross (to w1118?). In Figure 1F NSC are labeled with DPN (gray), neurons with elav in the pros-RNAi but for the brat-RNAi the legend suggest that Ase and Pros were used and this needs to be explained in the legend. In Figure 1G it was not clear what the side panels were indicating for each panel.

We have precised the genotypes, including control in the legends of now Fig. 2. We have also explained the side view and what the different molecular markers stained for directly on the figure as well as in the legend (*e.g.*, Ase (Type I NSC + mINP)).

Figure 2 the cartoon outlining the approach was useful and the data very clear on the effect that overexpression of PTEN had. For the statistics in Figure 2C, this was presumably a non-parametric test and should be outlined as such. For these sets of crosses what was the control? This experiment represented a very powerful approach using two expression systems to manipulate the NSC and the CG at the same time. What was the QF2 driver used in this experiment (cyp4g15-QF2?). The description of the QUAS-PTEN transgene was clear but how the QF2 driver was constructed was not…either include the details or reference the prior work that established this line.
For the panels in B it would have been nice to have a dashed line around the CG chambers in the digitally magnified panels to emphasize the continuous boundaries.

- We are sorry we forgot to add the test for this figure (now Fig. 3C). As we switched from raw to percentage of non-encased NSC lineages, the statistical test has now changed to a generalized linear model, what is stated in the legend.
- We have now precised in the legend the use of the Q and GAL/UAS systems as well as the drivers. However, we think that this experiment is an example of complex genetics, so we have chosen to keep the exact genotypes separated. All the detailed genotypes and conditions of all experiments have however been grouped in Supplemental Table 1, which we hope will make them more accessible.
- *cyp4g15-QF2* has been described previously (Rujano et al, 2022), and we have now added the line and reference in the method.
- We have now delineated two representative CG chambers representing individual encasing of NSC lineages using a white dashed line in Fig. 3B.

Figure 3 investigates the presence and roll of Drosophila Ecad (shotgun). For panel B, was there no neurons in this region as there seems to be no elav staining. Could the same yellow arrowheads and asterisks be used in B to highlight the same features? In panel 3C what was the control cross?

• We have added a figure (Fig. 4B) with a neuronal staining for Armadillo, and used asterisks for the NSC as we did for Shg.
• The control cross is *Nrv2::GFP, wor-GAL4; tub-GAL80^{ts}* x *w^{1118}*. It is now precised in the legend and also detailed in Supplemental Table 1. The same control line was used for *shg*, *nrx-IV* and *nrg* knockdown, and we decided to illustrate it in picture only once for the sake of space (while quantifying the corresponding control each time for each experiment).

Similar to above, dashed lines around the CG chambers might help in the higher resolution image or perhaps focusing on a smaller region at higher digital magnification so that the integrating of the lineage separate can be more easily be seen.

• To follow the same principle as in Fig. 3B, we now have delineated two representative CG chambers representing individual encasing of NSC lineages using a white dashed line in Fig. 4E.

For panel 3F, the encasement of the individual lineages was harder to see. The data suggests that there was some degree of breakdown of the chambers. Here a dashed line indicating the boundaries would be useful as well as a higher digital magnification as it was hard to see when lineages were mixed or not.

• In this condition, we did observe a number of NSC lineages not individually encased, albeit in a small number. For the close-up with higher magnification, we choose to show one of these occurrences rather than the behaviour of most of the population in order to nuance our conclusion on *shg* requirement. As such few proper individual encasings can be seen on this magnification, as noted by Reviewer 2.
We think outlining all the lineages would make the panel hard to read. We have now delineated two representative CG chambers representing individual encasing of NSC lineages using a white dashed line, and used yellow dashed lines for non-individually encased NSC lineages. This colour code is now used for the other genotypes.

Three shg-RNAi lines are listed in the methods but only one line was used for the data analysis and the arm-RNAi was not used (even though it was listed in the materials). Could the authors please list the RNAi used in the actual experiment and if others were used and what phenotypes were associated with those lines.

• We have double-checked the table for transgenic lines used in the Methods.
• We have quantified the level of knockdown for the lines we used (Supp. Fig. 4D-E) and precised for each experiment which RNAi line was used.

Figure 4 represents the key finding of this manuscript and presents compelling data for a role of NrxIV and Neuroglian. For this data set the lineage tracing images were the most compelling. Here the authors used the white dashed lines to great effect to indicate the CG chamber boundaries, though again a higher digital magnification if possible, would have shown the mixed lineages better. Please specify what the control cross was for these experiments.

• We have now dedicated a whole figure to *nrx-IV* (Fig. 6) and *nrg* (Fig. 8). This should help with displaying the pictures, while keeping the same absolute crop size for the close-ups.
• For simple experiments (Fig. 6B), we have added the genotypes. For more complex genetics (Fig. 6D), they are detailed in Supplemental Table 1.

For the quantitation of these experiments, it was unclear what constituted a positive score for not being encased individually. Comparing the images in Figure 3F there are clones that appear side by side without membrane between or with membrane processes that extend between but do not full encompass each clone. Yet Shg was classified as not playing a key role.

• We have now detailed more in the Methods how we scored encasing (Methods lines 246-265). Individual encasing means finding only one colour for Raeppli-NLS per CG chamber (for NSC lineages scored by Raeppli induction), and one NSC/Dpn$^+$ cell per chamber (for NSC themselves). It has to be done in 3D (through the z-stack) to be as accurate as possible.
Of note, we are conscious that getting an absolute, exact quantification for such data is extremely challenging and painstaking, due to the complex 3D architecture. For Raeppli-NLS also exists the possibility that two clones with the same colour might be induced next to each other, and are as such indistinguishable. We did the best we could, yet that is why we think the qualitative/semi-quantitative aspect of the phenotype is also very important to represent, and do not change the core interpretation for *shg*, *nrx-IV/wrapper* and *nrg* phenotypes.
• For original Fig. 3F (now 5B), as mentioned earlier, we decided to display one of the rare (yet clear) occurrences we found of non-individual encasing to nuance our conclusion on *shg* requirement. We are convinced that *shg* is not required for individual encasing in normal condition, as supported both by RNAi and mutant data. However, we found it harder to conclude regarding its role when CG growth is temporarily blocked. We did find a statistical difference compared to just blocking growth, suggesting *shg* might be some sort of back-up mechanism. Yet the percentage of NSC lineages affected still stay very small. Moreover, the genotypes start to be very complex, and might non-specifically bias the system towards more fragility/delay. As such we wanted to be cautious and say we could not exclude a role of *shg* as a safety mechanism. We have made this clearer in Results lines 177-180.

Looking at Figure 4G the same glial processes can be seen between lineages and there are examples where individual lineages are segregated. The authors suggest that the Nrg-RNAi resulted in CG membrane that were "broken" or "irregular" but these could also present CG membranes that had or were in the processes of extending processes around the NSC.

• The choice of “broken” or “irregular” was not inferring the origin of such phenotype (*i.e.*, lack of growth or post-growth alteration), but what we found best to describe what we saw. We changed this description, hoping it to be less excluding: “CG membranes rather seemed irregular, exhibited gaps and broken/stunted ends”.

The statistics also might be more compelling if the ratio of "encased" to "non-encased" was reported, rather than reporting the raw numbers of "non-encased" given that different numbers of VNCs and likely different numbers of lineages were scored for each genotype.

• For the ratio, we did not think it was required as the different lines have a similar number of NSCs per VNC (Supp. Fig. 10A-B), and we represented the number of non-encased NSC/NSC lineages per VNC (and not total across all VNCs scored). Nevertheless, we agree with Reviewer 2 it is an easier representation for the readers. We have thus changed all our encasing data as “Percentage of non-encased NSC/NSC lineage” and adapted the statistics accordingly (using a generalized linear model).

Figure 5 tested the potential partners for NrxIV and Nrg in mediating adhesion. This figure was less compelling than Figure 4 in that the effects of wrapper RNAi or changes to Nrg levels were hard to visualize.

• We have split previous Figure 5 in two, each part now focusing on one of the adhesion complexes (Wrapper on Fig. 7; Nrg on Fig. 9). We have now provided a clearer picture of the phenotype in *wrapper* RNAi, also showing the orthogonal view (Fig. 7B). We are not sure of what Reviewer 2 means by “changes to Nrg levels” on the original figure?

Wrapper-RNAi in the CG generated the same large chambers phenotype observed during Nrx-IV knockdown in the NSC. However, the powerful approach of lineage tracing utilized in Figure 4 could not used here making it hard to be sure that lineages were not separated. The author state "Moreover, this interaction is essential to produce individual encasing of NSC lineages by CG, implying that in

absence of such recognition, CG cannot properly recognize and individually sort NSC lineages". Without the lineage tracing the authors might want to qualify that statement somewhat.

• We have now added and quantified lineage tracing data for *wrapper* RNAi using the following cross
Dpn-Frt-STOP-Frt-LexA; ; cyp4g15-GAL4, LexO-Raeppli-NLS
X
yw, hs-FLP; Nrv2::GFP, tub-Gal80^{ts}/CyO; UAS-wrapper RNAI
yw, hs-FLP; Nrv2::GFP, tub-Gal80^{ts}/CyO;
These data are found in Fig. 7C-D.

The lack of phenotype when Nrg was knockdown in the CG suggests that there is no homophilic interactions between the CG and the NSC. Or conversely that the knockdown in the CG was less effective. For the Nrg-RNAi line used was this line equally effective for both isoforms?

• We have quantified *nrg* knockdown data showing the loss of Nrg signals for all Nrg isoforms (Nrg::GFP, Supp. Fig. 8A-B; mean of 0.50 normalized to control), Nrg^{180} (BP104 antibody, Supp. Fig. 9C-D; mean of 0.10 normalized to control) and Nrg^{167} (Nrg^{167}::GFP, Supp. Fig. 9E-F; mean of 0.25 normalized to control). The *nrg* RNAi line seems effective for both isoforms. The difference might be due to the stability of GFP fusion proteins (both for Nrg::GFP and Nrg^{167}::GFP), while Nrg^{180} is directly assessed with an antibody. This is discussed in the text Results lines 384-390.

Misexpression of Nrg180 in CG altered CG morphology and it was suggested a loss of encasing of NSC lineages (as did Nrg167 and a Nrg with a GPI tag suggesting an increased in homophillic binding in the CG). However, the effects of Nrg167 were far far stronger than that of Nrg180. The Nrg167 CG appeared rounded with little or no processes, so much so that a higher resolution image was not provided. Given this whole sale change in glial cell morphology, this does not exclusively suggest a increase in "CG to CG adhesion" through increase Nrg homophilic interactions but rather there are many other potential explanations such as a change to CG survival or differentiation. The quantification of the non-encased NSCs would be more compelling from the Nrg180 as the CG in that line appear to more be less disrupted and less morphological disrupted.

• We agree with Reviewer 2 that we cannot exclude a number of explanations for the striking phenotype associated with Nrg^{167}, including strength of the line itself or different signallings through the C-terminal part. CG features might also be affected by the overexpression *per se*, but also as a secondary consequence of a forced/altered adhesion (NSC encasing might very well give some feedback on CG growth, whose control is not known yet), two options we cannot distinguish between. Yet, the primary effect of Nrg overexpression is to enrich CG with molecules having a high affinity for strong homophilic interactions. As such, we believe that these data have to be taken within the context of classical tests for differential adhesion, without excluding further interpretation.
• We have now:
1. added the effect of expressing Nrg^{GPI} in CG, what produces the same phenotype as Nrg^{167}, and which implies that adhesion alone causes this phenotype.
2. quantified of NSC encasing for Nrg^{167}, Nrg^{180} and Nrg^{GPI}.

One quick note that for figure 5E (and the supplemental) - this figure would be more compelling if the blue channel was changed to magenta as the blue was very difficult to see.

• We have now used a lighter blue when representing Nrg^{180} (and yellow for Nrg^{167}).

Figure 6 was a very clear demonstration that the loss of NrxIV and Nrg lead to changes in axon projections. This experiment made elegant use of the Raeppli CAAX tag to label the cell bodies and axons. For the methods it appears that RNAi expression was continuous from ALH0 through to 72 hours later. The authors used wor-GAL4 and it would greatly help the interpretation of these experiments to know how long this driver persists expression in progeny of the NSC. For instance,

would all progeny still be expressing the RNAi for a considerable time or would the differentiated neurons no longer experience knockdown of NrxIV and Nrg. An explicit statement of this would help the reader understand the significance of the results in both figure 6 and 7. Otherwise, if the GAL4 and the RNAi expression persists, the conclusion that the loss of NrxIV and Nrg adhesions in the NSC influence the extension of axon tracts should be qualified given the roles that both proteins might play in axon pathfinding. In particular Neuroglian has a known role in axon pathfinding and thus changes in axon projects could reflect the loss of these functions.

• We completely agree with Reviewer 2 that the effect of *nrg* and *nrx-IV* knockdown on axonal features (and further locomotor behaviour) could be from a role of these molecules in the neurons, and not in the NSC *per se* (or coming from their role for niche formation exclusively). That is why we tried to keep our conclusions generalized to NSC lineages rather than NSC (hence our use of the lineage clonal analysis and of "L^{NSC}"), and to not causally link niche formation and axonal/behavioural features by centering back on the adhesions themselves rather than their other functions (*e.g.* title/conclusion such as: "Nrx-IV and Nrg adhesions are required in NSC lineages for correct axonal path during development"). We are sorry if it did not appear clearly.

1. Regarding *wor-GAL4*, it clearly perdures in the progeny, to which it is passed down through division. It is known in the field, and our own data on RNAi knockdown (previous Supp. Fig. 4C-D; 5C) showed that protein staining is decreased in neuronal progeny, indicating that RNA interference is also passed down to GMC and neurons. That is why we called/used *wor-GAL4* as a driver for NSC lineages (L^{NSC} = NSC + progeny for some time) during development and did not say it is exclusive to NSC.

To make this very clear to the reader we have now:

• documented in Supp. Fig. 1B the expression pattern of *wor-GAL4* in L3 (driving myr::mCherry from ALH0 to ALH72 through tub-GAL80^{ts} control), showing it covers NSC, neurons and axons.

• showed in Supp. Fig. 1C that RNAi knockdown driven by wor-GAL4 is efficient throughout the expression pattern of the driver, displaying side by side picture of *wor-GAL4 > UAS-myr::mCherry + GFP RNAi* and *wor-GAL4 > myr::mCherry + mCherry RNAi*. While it does not recapitulate what is exactly happening for all the adhesion proteins, as specific RNA and protein lifetime/stability will also have an impact, we believe it still gives a general frame.

• added data for *nrx-IV* and *nrg* knockdown under *nSyb-GAL4* (Supp. Fig. 6E and 8C), which drives exclusively in mature neurons (so primary in larva). In these conditions, we do not observe any CG phenotype.

• decided to keep *wor >* rather than *L^{NSC} >* for labelling the different genotypes in the figures

2. We have checked that our conclusions were on NSC lineages and not NSC throughout the text.
3. We have refined our summary schematic (now Fig. 12) to ensure our conclusions on niche formation, neuronal features and behaviour were understood as separated.
4. We have reinforce the distinction between functions in Results lines 442-443.

For Figure 6, a more compelling data set might be to show changes in specific axon projections as a consequence of the RNAi knockdown. Given that Raeppli was used couldn't some specific markers for different subsets of neurons be utilized to better indicate changes, such as FasII or FasIII axonal markers for instance?

• We think the use of Raeppli is powerful as it shows an alteration of axonal projections throughout the VNC, and not tied to a specific lineage.

• Nevertheless, we agree tracking specific lineages would reinforce our conclusions. We have added data for FasIII lineages (Fig. 10G-K), showing that FasIII level/localization is altered under *nrx-IV* and *nrg*, but not *shg*, knockdown in NSC lineages.

Figure 7 shows that loss of NrxIV and Nrg in the NSC lineages has resulting effects on locomotion in adults in particular leads to hyperactivity. Given the changes to axonal projections in Figure 6, it wasn't too much of a surprise that there might be some behaviourial changes given the degree of disruption. However, this section seemed oddly out of place and it wasn't very clear how this related to the changes in the CG chamber formation versus the whole sale disruption of axonal projections. The use

of a cartoon to illustrated the gene manipulations (similar to early figures) would really help in this figure to follow the genetic manipulations utilized and to give the reader a very clear idea of when the RNAi knockdown was occurring and when it ended.

• Our rationale was linear. We first focused on characterizing the role of different adhesions in the structure of the niche, what constitutes the core of this paper. We then could not but ask their functional relevance to neurogenesis, linked to or independently from their function in niche formation. We first tested NSC parameters and did not find major changes. We then moved to the other potential cellular target, the newly produced neurons. Reading previous literature oriented us towards their primary axonal projections, which we tested. We then wanted to test not only neurons' structural features but also functions. As secondary neurons produced during larval stage need to mature during the pupal period and are functional in adults, we had to test it at this stage.
• While we agree with "Given the changes to axonal projections in Figure 6, it wasn't too much of a surprise that there might be some behaviourial changes given the degree of disruption.", we still think that our findings bring something exciting, and still not fully predictable. While axonal/neuronal defects have been documented in a number of conditions during embryonic or larval development, there are actually very few studies (close to none) which checked whether it had any importance later in the adult. Rescues are not rare during development, and in truth we find it interesting that altering these adhesions only during larval stage has such an effect in adults, while an important remodelling and further axonal layering is taking place during the pupal period.
• Regarding the "it wasn't very clear how this related to the changes in the CG chamber formation versus the whole sale disruption of axonal projections", we indeed did not (and could no) conclude on this part. We stated that disrupting adhesions in the niche during development affects niche structure and adult behaviour, without bringing causal link between the two (as distinguished in the title). In the discussion, we proposed what might link, or not, the two.

• We now have:
1. precised our rationale in the text (Results lines 515-525), explaining that the function of secondary neurons has to be assessed at the adult stage and that causally linking developmental defects to adult health is an important step.
2. added sketches to explain the timing of our knockdown (Fig. 11 and Supp. Fig. 12), showing that only knocking down the different complexes during larval stages has such effect in the adult.
3. made clear distinction between the role of adhesions in niche formation versus neuronal and behavioural features, in the text (Results lines 442-442 and 590-598) and also in Fig. 12
4. yet, added behavioural data for *wrapper* RNAi (Supp. Fig. 12 and Results lines 569-589), which allow us to propose a link between niche properties and adult behaviour via this specific adhesion.
We hope these additions will clarify the message we would like to convey.

Minor:
The use of the different RNAi lines - it would be nice to include references in the table to papers that have previously used and verified these lines as being effective.

• We have added the quantifications of knockdown efficiency for the RNAi lines we used for *shg*, *nrx-IV*, *wrapper* and *nrg* (Supp. Fig. 4D-E, 6B-C, 7A-B, 8A-B and 9C-F).

Some abbreviations needed to be defined…for instance ALH24 etc.

• We have checked throughout the text what we had missed.

For the statistics, please outline what do the box and whiskers represent…the min/max or the 95% percentile and is the mean or median shown?

• These were explained in the Statistics part of the Methods to avoid repetition (Methods lines 331-335; now lines 371-374). We have also brought them in the legends.

Reviewer #3: This manuscript by Banach-Latapy et al addresses the question of how the structure of larval neural stem cell (NSC) niches is kept together. It builds on the notions that the NSC is encased by glial processes, that neural circuits are organised on cell-lineages and that the structure of cell lineages set up in early development determines the ultimate structure of adult neural circuits and therefore impacts behaviour. They test the involvement of various known cell adhesion molecules for their involvement in adhesion between NSC and their progeny ganglion mother cells (GMCs) and neurons, and between NSCs and surrounding cortex glia (CG). The main claims are: (1) Neuroglian is involved in homophilic interactions between NSC and niche progeny cells; (2) Neurexin-IV and Wrapper are involved in interactions between CG and NSC lineage cells; (3) disruptions to these adhesion mechanisms disrupts fasciculation and (4) adult behaviour. The microscopy is on the whole very nice and the identification of distinct cell adhesion mechanisms for intra-lineage and neuronal-lineage to glia interactions will be of interest to scientists focusing on these NSC lineages. The work has some important weaknesses that need addressing, and below are suggestions for improvement.

1. The manuscript text is difficult to follow, verbose, the results section is too speculative, and the narrative is too lightly linked to the evidence provided. The lack of a direct link in the text to the experiments makes it hard work to compare claims against the scientific evidence.

• We note and understand that Reviewer 3 does not like our writing. We have changed specific points we agree were too vague, incorrect or with an agency meaning (see further responses to specific points). As part of this judgement is subjective and linked to personal preference, and has to be balanced with other positive comments, we still have kept our general organization.
• The figures and the experiments they represent were all referenced in the text and followed by their interpretation and conclusion, using a classical way to organize a Results section ("title summarising the main conclusion > question > methods > interpretation > conclusion > transition to next question" for each chapter).

Furthermore, it is also difficult to understand the figures, as the labelling is very minimalistic or absent (e.g. no GAL4 drivers, no antibodies, no reporters, etc), so it is very hard to dig out the experimental source of the images provided. Even the figure legends lack some genotypes and necessary experimental details -nowhere in the paper are full genotypes provided - and figure legends lack a statement on findings and how they relate to the claims, making it rather hard to link the data in the figure to the claims in the text. It ought to be possible to understand a paper by reading the figures, and here it is very hard work. Table 1 is a helpful summary given the context above, but if the text, figures, figure legends and genotypes had been clear, this table would have been un-necessary. Thus, text, figure labelling and figure legends must improve to demonstrate that the evidence provided supports the claims.

• We understand that the legends were found to be too minimalistic, and we agree we missed some useful specific details. Part of the original choice was motivated by the desire to help non-Drosophilist readers (which we think would little benefit from the precise description of complex fly genetics) and to focus on the message, while the technical details were left in the Methods for specialists. Indeed:
- Full complex genotypes (Raeppli, Coin-FLP, MARCM) were provided in the Methods (lines 218 to 279 and 323 to 329), also including exact regimens of induction and culture.
- Some of the simpler genotypes and regimens were still written in the legend (for exemple, previous lines 75-76: "L^{NSC} *> shg RNAi*, driver line *Nrv2::GFP, wor-GAL4; tub-GAL80*^{ts}). Larvae are dissected after 68 h at 29°C from ALH0.")
- The figures themselves displayed the cellular or molecular markers observed, and the legends precised the tools (reporter; antibody) used. See as example for original Fig. 1C, a description we standardized throughout the legends: "Confocal pictures representing the dorsal region of the larval CB and ventral region of the VNC at ALH72 (at 25°C) labelled with markers for the CG membranes (*Nrv2::GFP*, green), glia nuclei (anti-Repo, yellow), NSC (anti-Dpn, grey) and neurons (anti-ElaV, magenta)."

• We are happy to find a working compromise between information and message, and we changed the following points:

1. We removed the simplification of the driver (CG > ; L^{NSC} >) and stated the enhancer used (*cyp4g15*; *worniu*; *ElaV*; *nSyb*).
2. We have completed the legends for simpler genotypes, including the control used.
3. To avoid having to try finding the genotypes and conditions in different places, we have now regrouped all the detailed genotypes and regimens in Supplemental Table 1, in the hope it will be easier to access.
4. We used a double nomenclature on the figure themselves, to help associating molecular markers with cellular features (*e.g.*, Nvr2::GFP/CG membrane).
5. We have brought back from the Methods the description of the whiskers and box plots.
6. We have completed some description of methods and conditions.

• Of note, Table 1 was mostly made to help putting the different phenotypes in the context of a differential adhesion hypothesis (rather than being a mere summary), something which was suggested by a specialist of this field. As such, we believe it is useful in this context to keep it.

The discussion is too long, verbose and speculative. The paper would improve if they reduced speculation throughout the results and discussion sections, and they focus on what the data show.

• The discussion is not only a summary of the findings but also the place made for the reader to think about their multiple aspects, including their meaning in the context of previous literature, their potential discrepancy and the perspectives they open. It is food for thought, and a welcome place to speculate.

• Our paper touches a number of very different aspects, which we thought would be good to also cover in the discussion. We would welcome feedbacks from Reviewer 3 on which parts of the discussion are not useful and could be cut to shorten it.

2. The text contains claims and generalisations not supported by the evidence provided. For example: the claim "lineage information prevails over cell identity" is a rather obscure statement not supported by data. No experiments have been carried out using cell fate markers to test cell identity, so no claims on cell identity can be made. The fact that Raeppli labelled distinct lineages in controls, prosRNAi and bratRNAi does not reveal anything on cell identity or cell fate, as cell fate within resulting tumours was not tested. Similarly, claim "CG are able to distinguish between lineages" has not been directly addressed, as no manipulation has been carried out to alter lineage cell fate.

• We understand that this statement "lineage information prevails over cell identity" is difficult to grasp. We meant to say that CG keep cells from the same lineage together, regardless of their identity and that the NSC identity is not sufficient to trigger individual encasing. In the same fashion, “CG are able to distinguish between lineages” was used to describe the observation that CG will keep all NSC lineages separated from each other, regardless of cell identity within the lineages and timing of NSC encasing itself. We have now removed these statements and rephrased the results (Results lines 44-45).

• We do not agree that “No experiments have been carried out using cell fate markers to test cell identity, so no claims on cell identity can be made” and that “no manipulation has been carried out to alter lineage cell fate”:

1. ElaV, Dpn, Ase and Pros are all cell fate markers and define, with other factors, neuronal, NSC, Type I NSC and GMC/neuronal identities. As such, we did test (previous Fig. 1F and current Fig. 2A-B) the identity of the cells in normal conditions and under lineage manipulations and found that changes in cell identity within a lineage did not prevent its individual encasing as a whole.
2. The different tumours we tested are all by principle alterations of lineage cell fate, as the lineage is not directed to differentiation but reverted to/maintained in a stem cell-like fate. *pros* and *brat* tumours both come for the knockdown of fate determinants for GMC or INP. *dpn* overexpression leads to GMC de-differentiation. Membrane-tethered aPKC prevents asymmetric division and the generation of the GMC fate. All these tumours are well-known examples of cell fate alteration in NSC

lineages, and cell fates (which we stained for) within these tumours have been thoroughly documented (reviewed for example in Homem and Knoblich, Development, 2012; Li et al, Bioscience Reports, 2014). For all these reasons, we do not think our conclusion were overstatements.

3. The distinct choice of methods at different points should be justified, explaining the rationale for choosing them in each case, e.g. why use Raeppli, CoinFlip, MARCM in each case.

• We have precised better why we choose Raeppli (Results Lines 27-31), COIN-FLP (Results Lines 87-92) and MARCM (Results Lines 139-143).

4. Given that the shg data (Figure 3) are mostly negative results, they could be dealt with more briefly in the text, or even moved to supplementary.

• We do not wish to follow this suggestion. We believe these data should be visible due to the literature context, as previous papers using a dominant-negative form of Shg reported strong CG phenotype, something we do not see with our RNAi and mutant approaches. The idea that Shg has a role in CG structure (and NSC proliferation) is the current knowledge in the community, and our own data call for a more nuanced conclusion. We actually did test Shg contribution very thoroughly because of these previous results.

5. Most phenotypes are not quantified, and they need to be, providing the sample size, penetrance of the phenotypes (frequency) and the expressivity (severity) if variable. This affects data in Figure 1 (all), Figure 2E, Figure 3A,B,C,H, Figure 4A,B, Figure 5A,B,C,E,G,H. The penetrance and sample size must be added either to figure or figure legend. Furthermore, Supplementary Figure 2B is not meaningful without quantification.
• All the lineage tracing (Raeppli-NLS) data, the axonal data, the NSC data and the behavioural data were all quantified, with sample size (n) and expressivity (given by the distribution of the data and individual values).
• For *shg*, *nrx-IV* and *nrg* RNAi phenotype on encasing, our choice was to offer a progressive lecture:
i) A qualitative yet representative illustration of the architectural phenotype using markers for NSC and CG membrane, to give to the reader a striking, simple and graspable view of the changes in CG.
ii) A quantification of these same knockdowns using a lineage tracing method, a way we found more accurate and powerful as we were interested in whole lineages and not only NSCs (an approach recognized by Reviewer 2's comments for Figure 5). For these, the expressivity is given by the Box-and-Whiskers plots. In addition, we indicated unusual variability (*nrg RNAi*, see Results Lines 273-275 and Figure Legends Line 144 in the previous version). Sample sizes (n) were indicated in the legends.
We quantifed i) only in absence of ii).
• We agree that we did not provide and have now added:
1. Penetrance. We originally did not comment as it was always 100%; we have now clearly stated it in the Methods, section Statistical analysis and reproducibility Lines 375-377.
2. Statistics for *wrapper* RNAi. We have now added lineage tracking data with Raeppli-NLS, which we quantified (Fig. 8D-E)
3. COIN-FLP statistics, in Fig. 3F and Supp. Fig. 3C.
• For tumour induction (previous Figure 1), we do not think quantifications are fully relevant, as it serves as a proof-of-concept that cells of one given lineage are encased together regardless of their identities. How many tumours are formed is not what we assess (and a different question on the technical and biological efficiency of transformation,) but what happens within each tumour. We still have provided data for *brat* tumours (Supp. Fig. 2C-D)

6. Claims: "CG are completely interchangeable", "This shows that any given NSC lineage can recruit other CG cells.." are not supported by data. The fact that loss of CG does not reveal a break in the membrane network does not demonstrate that 'cells are interchangeable' nor that "any given NSC can recruit other CG cells'. These claims must be removed or new experiments must be carried out to provide evidence.

• We understand this statement is too strong, and we have rephrased it (Results lines 84-85 and 102). Nevertheless, a clonal analysis is a generalization, as one infers population behaviour based on its sampling by many clones. That is what we did in Fig. 2, for which we agree we should have given the quantifications to justify it. There are now found in Fig. 3F and Supp. Fig. 3C.
• Our recent paper (Rujano et al., 2022; referenced) shows that the membrane of one CG cell fully enwraps individual NSC lineages. As such it hard to imagine how preventing the growth (PTEN) (or killing it, Reaper) of one CG cell would not leave a clear break in the CG membrane if other CG cells did not replace this lost encasing (and as such are recruited by NCSs). Of note, the same conclusion and vocabulary on filling gaps and recruitment was reached by a previous study on CG plasticity around mature neurons (Coutinho-Budd et al, Genes&Dev, 2017), what we did mention (reference 39 now).

7. a) RNAi experiments: no evidence has been provided that any of the RNAi lines can knock-down gene expression. RNAis well known to yield variable results, and the standard approaches to deal with RNAi are: (1) use multiple, distinct RNAi lines to confirm that similar phenoytpes are observed; (2) test with qRT-PCR for real lowering of mRNA levels, or antibodies could also be used when available to test protein knock-down; (3) and combine RNAi with heterozygous mutant background to reduce phenotypic variability. The authors should validate their data with at least some of these approaches, including either 1 or 2.
•. We did provide representative qualitative data showing the effect of RNAi knockdown at the level of Shg, Nrx-IV and Nrg (plus the Nrg^{180} isoform) in Supp. Fig.: 3D; 4C-D; 5C, using antibodies' staining as Reviewer 3 mentions. These data showed that all the RNAi lines we employed worked.
• We have now added the quantification of these already existing data (Supp. Fig. 4D-E, 6B-C, 7A-B and 9C-F), and added quantification for the effect of *wrapper* RNAi on *wrapper* mRNA (Supp.Fig. 8A-B).
• We also show that we recapitulate *nrx-iv* (Supp. Fig. 6D) and *wrapper* (Supp. Fig. 8C) phenotype with other RNAi lines.

b) In this context, the authors could not recapitulate with shg-RNAi and null clones previous findings published with shgDN. As they did not provide evidence that RNAi is working, the published data cannot be invalidated.

• As stated above, we did provide representative qualitative evidence that *shg* RNAi was working (for 3 transgenic lines, Supp. Fig. 3D; now quantified). Moreover, we backed up these data using null *shg* mutant clones (in which the loss of Shg signal was also checked for extra-safety, previous Supp. Fig. 3F, now Supp. Fig. 55A), and this genetic approach cannot fit the issue with the RNAi not working. To summarize, we used two independent approaches (RNAi and null mutant clones), both checked for knockdown, and found there was no alteration of CG architecture, contrary to previously published data using a dominant-negative construct. As such, we do not see a reason to downplay our conclusions with these tools.
• We did not write that the previous data should be invalidated, but rather proposed in the Discussion (previous Discussion Lines 35-39, now Lines 33-38) a balanced view including a number of explanations for this apparent discrepancy.

8. WrapperCRIMIC-GAL4 line was used to visualise wrapper expression driving UAS-mCD8GFP and UAS-HisRFP, co-stained with anti-Repo. Crimic lines carry 3xP3-RFP which is expressed in all glia, and the expression is so strong that it is detectable in both 488 and 546 channels without staining. Thus, if still there, the expression does not necessarily represent wrapper expression, but the 3xP3 promoter. Unfortunately, removing 3XP3 from Crimic lines, is not trivial. Did the authors remove 3xP3-RFP from the crimic line? If so, they must provide the evidence. If they did not, these data should be removed.

• We agree that the 3xP3-GFP of the CRIMIC lines is expressed in glial cells, at least partly in the CG. However, this cannot explain the nuclear, neat His::mRFP signal, which co-localises with Repo, a marker for glial nuclei (previous Supp. Fig. 5A) and thus which can only come from the CRIMIC

enhancer. Drosophila glia have a layered organization, and CG are the ones found around NSCs. As such, we believe that our data, with His::mRFP$^+$, Repo$^+$ nuclei around NSCs show that the *wrapper* CRIMIC line drives in the CG.

• We nevertheless understand Reviewer 3's issue about the contribution from the 3xP3 promoter. We have added data showing *wrapper* RNA FISH overtime in normal conditions (Fig. 8A), and compared to *wrapper* RNAi knockdown for ALH72, which we have quantified and in which midline expression of *wrapper* is conserved (Supp. Fig. 8A-B).

• We would like to note that these data were not alone in supporting Wrapper expression in the CG. First, we provided an antibody staining (previous Fig. 5A) with an antibody validated previously on midline glia and which can also detect Wrapper misexpression in NSC lineages (previous Fig. 5C). Second, two other papers (Coutinho-Budd, Genes&Dev, 2017 and Plazaola-Sasieta, Glia, 2019; both of which we referenced) had previously reported *wrapper* expression in the larval CG, using a *wrapper* enhancer identified by the Freeman lab. While the CRIMIC data provided additional support, and a temporal characterization, our conclusion on *wrapper* expression in CG did not solely depend on that.

9. Statistical analysis: there are problems with the stats. In figure 3G, Figure 7C,D legend says they used "OneWay Anova and Kruskal Wallis multiple comparison test". Instead, this should have been Kruskal Wallis Anova and post-hoc Dunn test comparison to a fixed control. However, both in Figure 3G and in Figures 2-5 (which use Mann Whitney tests), they have applied non-parametric tests for quantitative data to compare categorical data ('encased' vs 'not encased' lineages). Categorical data such as these are ('encased' vs 'not encased') require testing with Chi-square tests. Thus, the authors must revise their statistics for Figures 2-5. (Mann-Whitney in Figure 6 is fine; Kruskal Wallis Anova in Figure 7 are fine).

• The name of the test for Fig. 3G (now Fig. 5C) was indeed not precise. As the tests have changed to accommodate the use of percentage, it has been replaced by a generalized linear model (see below).

• Regarding Fig. 2-5, and after checking back again with the statisticians of the Bioinformatics Hub of the Institut Pasteur, we were right in using a non-parametric Mann-Whitney test for the encasing data. The encasing data are not categorical (*i.e.*, allocated property) but numerical (*i.e.,* counts) data, as we measure a number of non-encased NSC lineages per sample/VNC. Using the Chi-square test would be wrong, as it would pool all data assuming they all are independent, while events are not independent within each sample/VNC.

• Encasing data are now represented as percentages and not raw values, as suggested by Reviewer 2. We thus are using a generalized linear model with a random effect.

p values must be provided for all data sets.

• All p-values were, and still are, displayed on the graphs themselves.

10. One key claim is that Neurexin-IV and Wrapper are involved in interactions between CG and NSC lineage cells. However, this claim is based on knock-down or over-expression experiments that demonstrate that these manipulations cause phenotypes, but they do not demonstrate that Neurexin-IV and Wrapper function together across cell types. If published, what is the evidence that they can bind each other? It would be best if the authors could do experiments that reveal their functioning together across cell types, e.g. double-knockdown or epistasis.

Also, this claim depends on Wrapper being expressed in CG. However, Wrapper is well-known to be restricted to the midline glia, thus the images shown here could correspond to background instead. Further evidence must be provided that wrapper is in cortex glia, for example by testing that the anti-Wrapper signal is lost in CG>wrapper-RNAi whilst the midline glia signal remains intact; and they could also check in the Fly Atlas single cell RNAseq database how many cells express wrapper in larval VNC.

• We found that that Neurexin-IV is expressed in NSC lineages and that Wrapper is expressed in CG (see point 8). Moreover, and very importantly, their knockdowns in the respective cell populations lead not to any CG phenotype, but the same, very specific phenotype, with bigger yet definite

chambers. Finally, previous papers have demonstrated the direct interaction between Neurexin-IV in neurons and Wrapper in another glial type (Stork et al., Development, 2009). As such, an interaction between Nrx-IV in NSC lineages and Wrapper in CG was the simplest explanation we could put forward fitting all these data.

• We understand Reviewer 3's concerns about proving the interaction between *nrx-IV* and *wrapper*. We have now performed epistatic experiments by comparing the phenotype of double knockdown versus simple (dose-compensated) knockdowns, precisely comparing:

i) Nrv2::GFP, wor-GAL4; cyp4g15-GAL4, tub-GAL80^{ts} x UAS-wrapper RNAi, UAS-Nrx-IV RNAi
ii) Nrv2::GFP, wor-GAL4; cyp4g15-GAL4, tub-GAL80^{ts} x UAS-wrapper RNAi; UAS-mcherry RNAi
iii) Nrv2::GFP, wor-GAL4; cyp4g15-GAL4, tub-GAL80^{ts} x UAS-RFP RNAI; UAS-NrxIV RNAi

All genotypes produced the expected phenotype with bigger yet well delineated CG chambers. As quantifying encasing of NSC lineages is challenging to achieve without a lineage tracing tool, which was difficult to add in these genotypes, we decided to use CG membrane density as a proxy of encasing. We validated the relevance of this proxy beforehand (Supp. Fig. 7E).

In these conditions, and assays, we found that the double knockdown resulted in a decrease in CG membrane density compared to single knockdowns (Fig. 7E-F), indicating that *nrx-IV* and *wrapper* interact in this cellular context (see Results lines 288-307).

• For concern about Wrapper expression, see our response to point 8.

11. Whilst behavioural phenotypes of reduced sleep and increased speed were found when nrg was knocked-down, no evidence is provided that these phenotypes are related to rest of the data.

• All the conditions we tested in the locomotor assay were the same tested both for chamber formation, NSC and neuron/axon parameters. While the primary message of our paper centers on chamber formation, it is also an overall characterization of the roles of these complexes during larval development. As such, we believe these data belong to this paper.

• Our rationale was indeed linear. We first focused on characterizing the role of different adhesions in the structure of the niche, what constitutes the core of this paper. We then could not but ask their functional relevance to neurogenesis, linked to or independently from their function in niche formation. We first tested NSC parameters and did not find major changes. We then moved to the other potential cellular target, the newly produced neurons. Reading previous literature oriented us towards their primary axonal projections, which we tested. We then wanted to test not only neurons' structural features but also functions. As secondary neurons produced during larval stage need to mature during the pupal period and are functional in adults, we had to test it at this stage.

• We now have:

1. precised our rationale in the text (Results lines 515-525), explaining that the function of secondary neurons has to be assessed at the adult stage and that causally linking developmental defects to adult health is an important step rarely achieved.
2. made clear distinction between the role of adhesions in niche formation versus neuronal and behavioural features, in the text (Results lines 442-443; and 569-598) and also in Fig. 12

We hope these additions will clarify the message we would like to convey.

For example, their cell biology data are taken from the VNC, whereas sleep depends on the brain.

• The circadian control of sleep is indeed done by neurons found in the CB, however our behavioural phenotype is NOT a rhythm one (as shown by the maintenance of the stereotyped period equally under Light and Dark cycles, see previous Supp. Fig. 7, now Supp. Fig. 11). The phenotype is a change in the amount and quality (velocity) of locomotion, a motor behaviour controlled by VNC neurons. We used sleep at the time to represent the amount of locomotion as a classical, widely-used parameter whose definition is a locomotor one. Sleep in Drosophila is indeed defined as cumulated time of stillness for at least five minutes, and as such is an expression of locomotor inactivity (or to paraphrase, "prolonged periods of immobility are used as a proxy for sleep", Beckwith and French, Frontiers in Physiolgy, 2019).

• To avoid confusion, we have now represented lack of locomotion differently, using referenced Rethomics functions (Geissman et al, 2019) measuring:

1. the percentage of time moving across LD cycles as the fraction of time moving within 30 min intervals (Fig. 11C)
2. the global time moving per fly corresponds to a total moving over total time ratio (Fig. 11D and Supp. Fig. 11).
• Velocity was and is still a locomotor phenotype.

Furthermore, genes can have unrelated, pleiotropic functions at different time points and the authors state that both Nrg and Nrx-IV have multiple roles in the CNS, including in dendritic arborisation and axon branching. So, could RNAi in mid-pupa disrupt dendrite and axonal growth or affect adult expression levels?
• We performed RNAi knockdown only during developmental stage (see Methods, Behavioural analysis; Results lines 454-463). The samples were shifted back to 18°C (RNAi inhibition) at early pupal stage/after pupariation (meaning after 1 day maximum at 29°C from visualizing wandering L3). The "mid-pupa" in the text was an oversight from an earlier draft (when we still included one replicate shifted after 2 days at 29°C from wandering L3) and not in accordance with what we finally had and what was stated in the Methods. We are very sorry about the discrepancy and confusion between Methods/Text and the legends. We have now corrected it and better precised the timing in both text (Results Lines 539) and Methods (Lines 366-367).
• For each gene, we thus compared a condition in which RNAi was only allowed during developmental stage ("induced") to a condition where RNAi was never on ("non-induced"). As such, the only difference is the alteration of adhesions in the niche and NSC lineages during this developmental time (3 to 4 days at 29°C, followed by nearly 15 days at 18°C).
• We have now made this regimen very clear by adding Fig. 11B and Supp. Fig. 12A.

Further experiments could resolve whether these phenotypes are linked to the alterations in the NSC niche. For example, they could knock-down nrg, nrx-IV, shg and wrapper in the adult only, to rule out whether adult functions of these genes were contributing to the observed behavioural phenotypes;

• We do not think "knock-down(ing) nrg, nrx-IV, shg and wrapper in the adult only" would be informative and we are not sure what it means to address.
1. If the concern is that the temporal control of RNAi knockdown is not strong enough, leaking throughout pupal and adult stage, we should see the same result in induced versus non-induced conditions, what we do not.
2. It might well be that these complexes have also unrelated (and possibly overriding) roles in the adult, and this is what knocking down *nrg*, *nrx-IV*, *shg* and *wrapper* in the adult only would really assess, not the contribution to the developmental phenotype.

similarly, by knocking down in neurons only, to rule out functions in axons, dendrites, etc; testing with qRT-PCR that expression levels in the adult are intact; or testing with neuronal morphology that axonal and dendritic arborisations are intact.

• We understand that Reviewer 3 wants to identify the consequences of in pupa or adult of knocking down the different adhesions during development, which would explain the locomotor phenotype. Albeit we fully understand the interest, this does not change the conclusion that disrupting adhesion complexes during development results in altered locomotor behaviour in the adult. Whether there are (likely) relaying steps in between and their nature (such as dendritic arborization, neuronal tracts remodelled (or intact), feedbacks on later gene expression or even CG architecture in the adult itself), does not change the origin of the phenotype. We do not claim how adhesion in development results in altered locomotion in adult (something we discuss in the Discussion, and what should be a whole investigation by itself), but that it does. We identify input and output; relaying steps might be many and the subject of further, deep investigation.
• We also tried to keep our conclusions generalized to NSC lineages rather than NSCs (hence our use of the lineage clonal analysis and of "L^{NSC}"), and our message is that the lineage as a whole is crucial for niche formation. *wor-GAL4* drives in the whole lineage and we showed that we had a similar

phenotype at the level of the CG niche when *nrx-IV* and *nrg* were knocked down with *ElaV-GAL4* (Supp. Fig. 6E and 8C). As such, performing the behavioural experiment with *ElaV-GAL4* would not help distinguishing between a role of these complexes *per se* in the neuron or for niche formation. To avoid misunderstanding, we have now reinforced the focus on lineages and:

- documented in Supp. Fig. 1B the expression pattern of *wor-GAL4* in L3 (driving myr::mCherry from ALH0 to ALH72 through tub-GAL80^{ts} control), showing it covers NSC, neurons and axons.
- showed in Supp. Fig. 1C that RNAi knockdown driven by wor-GAL4 is efficient throughout the expression pattern of the driver, displaying side by side picture of *wor-GAL4 > UAS-myr::mCherry* and *wor-GAL4 > myr::mCherry + mCherry RNAi*. While it does not recapitulate what is exactly happening for all the adhesion proteins, as specific RNA and protein lifetime/stability will also have an impact, we believe it still gives a general frame.
- added data for *nrx-IV* and *nrg* knockdown under *nSyb-GAL4* (Supp. Fig. 6E and 8C), which drives exclusively in mature neurons (so primary in larva, outside of the NSC niche). In these conditions, we do not observe any CG phenotype.
- decided to keep *wor* > rather than L^{NSC} > for labelling the different genotypes in the figures
- checked that our conclusions were on NSC lineages and not NSC throughout the text.

• Yet, we now provide behavioural data for *wrapper* RNAi in CG (Supp. Fig. 12 and Results lines 569-598), which are very similar to what was obtained for *nrx-IV* RNAi under *wor-GAL4*. *Per se*, it demonstrates that altering CG properties during development (by removing *wrapper*) impacts adult locomotion. In addition, it suggests a link with niche architecture, as knocking down each member of a complex linking CG to NSC lineages, important for NSC encasing, and only during the developmental time of niche formation, results in a similar locomotion phenotype.

The sentence in the discussion line 137 "In our case, a reasonable explanation… translates to dysfunctional motor neurons in the adult" is not an explanation, but rather a tautology lacking in experimental evidence. Can they test why the niche and axonal phenotypes described resulted in increased activity (eg rather than decreased or other)?

• Not all axonal issues during development might equal dysfunctional neurons in the adult, due to potential rescue, compensation, remodelling and pruning in pupal stage, etc. While axonal/neuronal defects have been documented in a number of conditions during embryonic or larval development, there are actually very few studies (close to none) which checked whether it had any importance later in the adult. Rescues are not rare during development, and in truth we find it interesting that altering these adhesions only during larval stage has such an effect in adults, while an important remodelling and further axonal layering indeed is taking place during the pupal period. As such we think our results on behaviour were not necessarily expected.

• This sentence is in the discussion section where we propose hypotheses and future directions which will need to be demonstrated. We were always careful to display alteration of niche architecture and behavioural changes as two outputs with no demonstrated causal link.

• Testing "why the niche and axonal phenotypes described resulted in increased activity (eg rather than decreased or other)" is a whole new, lengthy study beyond the scope of this paper. To the best of our knowledge, hyperactivity is yet to fully understood at the circuit level itself.

If they can provide some further evidence, it would be interesting to keep behaviour data. However, the title should be modified, as the evidence does not support the claim that differential adhesion directly "controls behaviour".

• We showed that removing these differential adhesions specifically in the NSC lineages/niche, and during development only, is enough to alter locomotor behaviour in the adult, regardless of the relaying steps in between and other functions of these complexes. We believe that supports the title, in which again we were cautious to not link causally the behavioural and architectural outputs of adhesions.

• We do not think these data are detrimental to/remove anything from the paper, but rather are exciting. As we mentioned earlier, causally linking restricted developmental mechanisms (*i.e.*, specific

adhesion) to specific adult behaviour (*i.e.*, locomotion) is rarely done despite its importance. We believe it will be a welcome opening for the community, which we hope will pick it up for more neurobehavioural approaches.

• Finally, we believe the new *wrapper* data link niche properties to adult behaviour.

Minor:

* The text assigns agency to cells " CG are able to distinguish between lineages…", "taken care of", "managed to..". Cells have no agency.

• We did not intend to bring intentionality in the paper, some expressions were used to help describing some complexes phenotypes. We agree it was wrong and that we should have been cautious. We corrected the corresponding sentences.

* The relationship between PTEN and PI3K/AKT is hidden; instead of PI3K/AKT, Figures 2A and 3E should refer directly to the data and show PTEN ON/PTEN OFF.

• We wrote in the text that PTEN is an inhibitor (well-known) of the PI3K/AKT pathway, which it is classically used to block it. We used it for this function, not for being PTEN specifically (previous Results line 85-86). As such, we think it is valid to represent it as a mean to turn PI3K/AKT off, what has been done and stated in many previous studies in various tissues. We are nevertheless happy to use PTEN in new Fig. 3A and 5A.

* Sentence in line 62: "These results thus show that the information allowing CG to distinguish between lineages…" is rather obscure.

• We have rephrased this sentence.

* Line 87: "However the CG network itself appeared gapless around PTEN clones… CG were able to compensate for the loss…". There is no experimental evidence in support of this and thus should be removed.

• All NSCs are individually encased despite CG cells within the population being prevented to grow. We were not the only ones to report and describe such property as a compensatory, plastic event (Coutinho and Budd, Genes&Dev, 2017). Please see our response to point 6 and the novel quantifications we bring (Fig. 3F and Supp. Fig. 3C).

* Sentence 118: "We then wonderer whether such adhesion was a property of differentiating lineages.." is unclear.

We have rephrased it.

* Sentence 123: "It was not the case when they originated from different lineages..." Where are the data in support of this claim? Following statements are obscure.

Section from 123 to 132 "this suggests that adherens junctions… In line with our findings… Altogether these results…." is very speculative, without supporting data, in fact disproved later, and is best removed.

"A strong Shg staining was detected between NSC-like cells from the same lineage (Supp. Fig. 3A). It was however not the case when they originated from different lineages, which were separated by CG membrane. This suggests that adherens junctions are not only a property of differentiating lineage, but, importantly, are found between cells in an inverse correlation with the presence of CG membranes. In line with this finding, we observed a strong Shg staining between NSCs of the optic lobe, another type of neural progenitors [41] which are contained within one CG chamber (Supp. Fig. 3B). Altogether these results suggest that adherens junctions could be a mean of keeping lineages together, providing differential adhesion that would ensure stronger bond between cells of the same lineage than between lineages and CG, and functioning as a physical barrier to further enwrapping of individual cells by CG membranes."

• We have conclusions based on observations (Shg is detected between lineage-related cells contained within one chamber, and not at the CG interface between different lienages; Fig. 3A and Supp. Fig. 3A-B).
•This led to proposing another hypothesis to test, and the last sentence is meant as a logical transition to the next section, in which this hypothesis will be tested (and thus might turn out to be correct or disproved).

* Sentence 274 "a population seen.." is obscure
We have rephrased it: “The expressivity of the phenotype was variable, with a class showing nearly no individual encasing (around 100% of NSC lineages grouped with at least another one, Fig. 8E) and a class with a range of milder alterations (less than 50 % of NSC lineages grouped with at least another one, Fig. 8E), something we also noticed qualitatively in Fig. 8B.”

* Sentence 461 "We did not find comparing induced and non-induced relevant…". This sounds odd.
• We explained why in the text (Results lines 461-462: “We did not find comparing induced and non-induced relevant, as these two conditions relies on very different regimens of fly husbandry”). In particular, induced and non-induced flies develop under different temperatures, and a paper from the Hassan/Hiesinger labs has shown that developmental temperature affects adult CNS wiring and behaviour (Kiral et al, 2021). As such it is irrelevant, and misleading to compare flies raised at different temperatures (what happens here between induced and non-induced).

* The order of figures is random in places and should be tidied up e.g. Figure 3, Figure 4, Figure 5.
• The figures have been reorganized.

* The images in Figure 4D do not clearly show data in Figure 4E and a better selection should be made.
• As with all Raeppli-NLS pictures, this image was selected from a VNC displaying a percentage of non-encased NSCs close to the mean of the population. As thus we believe this image is representative and should be kept to ensure homogeneity.

* Magnification must kept consistent when comparing similar data types. Fer example, Figures 4D and G are of the same type, but of different magnification, and should be shown at the same magnification in order to compare the data. Same in Figure 5A and F. Etc, revise all magnifications.
• Magnifications were standardized depending on the type of data they show. For example, previous Fig. 5A is purposedly not the same as previous Fig. 5F as we rather want to compare it to previous Fig. 5C, 5G and Fig. 3H.
• We have revised previous Fig. 4G, now Fig. 8D, which was indeed at an incorrect magnification.

* The dash lines in Figure 4D,G prevent seeing the data and would be best removed from the green channel.
These dashed lines have been removed throughout the paper.

* They must explain in text and figures why they use conditional expression with tubGAL80ts in all experiments.
We have added a sentence in the Methods (Lines 138-144). We do not think that repeating this technical detail of *Drosophila* genetics would help the generic reader, as long as it is clear the experiment is conditional/temporally controlled.

* Blue channel in Figure 5E,F is critical and very difficult to see and would be best shown in white.
We have now used light blue for Nrg^{180} throughout.

* The alternating use of E-Cadherin and Shg nomenclature is rather confusing, one should be chosen and consistently used.
We have now used Shg throughout.
