# Supplementary material for: Differential adhesion during development establishes individual neural stem cell niches and shapes adult behaviour in Drosophila
Source: PLoS Biol. 2023 Nov 9;21(11):e3002352. doi: 10.1371/journal.pbio.3002352 (PMC10635556; doi:10.1371/journal.pbio.3002352)
Supplement: Attachment — Submitted filename: Response_Reviewers.pdf [file pbio.3002352.s018.pdf]

Response to reviewers' comments of manuscript PBIOLOGY-D-23-01796R1

In addition to the responses below, we are providing the Tracked change file to follow our exact changes.

New or updated figures are:
• Fig. 2E
• Supp. Fig. 2E-F
• Supp. Fig. 3C

Reviewer #1: The authors addressed all our concerns and suggestions in the revised manuscript. We support publication of this study.

We thank Reviewer 1 for his positive appreciation of the study.

Reviewer #3: The authors have made a considerable effort to improve the paper and have addressed several of my criticisms. There are many more quantifications, and the FISH and RNAi evidence that wrapper is expressed in CG are now compelling. However, other aspects have not been dealt with and will require improvement. The work is interesting and would have broad appeal for the PLoS Biology readership, the methodology appears to be rigorous and the microscopy is beautiful, but the interpretation of findings, the subjective narrative and conclusions lack rigour in places, and presentation of the evidence in support of conclusions needs to be improved. The following revisions are essential to support the current conclusions.

1. Text, genotypes, poor labelling, etc: some limited improvements have been made, but text and many figures still lack essential information to enable scientists reading the paper to verify the authors' claims against the data. Precise information should be provided in figures independently of the manuscript body text. Some labelling is good, some is not.

• We do not agree with the generalization that the improvements have been limited and that the figures cannot be understood with the legends provided. We will detail further why we think so.

For example, Figure 3 and Supp Fig 3: "Delayed chamber PTEN", "PTEN clones", "PTEN ON", lack information on whether these are for PTEN visualisation, PTEN over-expression, PTEN mutant or PTEN knock-down, in which cell type, or what type of clone. Figure legend has not been improved either, as for B there is no explanation, C says "PTEN conditional block", E "CG growth was blocked (PTEN)". "Blocking" is not a functional genetics term.

• "Block" was not referring to PTEN, but to cellular growth (see Previous Figure legends Lines 83-84), and we do not think it is an inappropriate term for such (it would indeed be for the gene). We have now made sure that it clearly refers to the cellular process and not the gene when we used it.
• Regarding PTEN, we have now changed both in the figure and in the legend the labelling for PTEN clones/overexpression. We have also remodeled the schematics of Fig. 3A and 5A to include the control condition and some simplified genotypes, which has been further included in the picture panels. We are grateful to Reviewer 3 for having pointed the need of consistency for this specific issue.

In Figure 2: "pros tumours" is unclear whether this corresponds to pros GOF, LOF, mutant or RNAi, nor with which driver. This should be specified as it is for other figures (eg SuppFig2D).

• This was stated in the figure legends:
"A-B) Adaptation of the CG network to NSC tumours. Type I *pros (Nrv2::GFP, wor-GAL4; tub-GAL80^{ts} > pros RNAi*) and Type II *brat* (*Nrv2::GFP, wor-GAL4; tub-GAL80^{ts} > brat RNAi*) tumours were induced and the organisation of CG membrane was monitored by *Nrv2::GFP* (green). Control line for both (*Nrv2::GFP, wor-GAL4; tub-GAL80^{ts} > w^{1118}*). For *pros*, larvae were dissected after 48 h at 18°C, followed by 2 h heatshock at 37°C and 48-52 h at 29°C. For *brat*, larvae were dissected after 72 h at 29°C. See Supplemental Table 1 for detailed genetics, timing, and conditions of larval rearing."
• We have now also put a simplified genotype on the figure.

In Figure 3, what exact timings are T1 and T2?

• We choose to add these details in both the methods and the full list of genotypes/conditions. We did not think it would help comprehension of the readers, as long as what happens at T1/T2 is clearly explained to support interpretation. We have now added them (T2 was actually already indicated by the time of dissection).

It should be possible to understand the data by reading the figures and figure legends, without reference to the manuscript text and the narrative. Currently it is not, and the text is often not explicit

either. The authors have provided a list of full genotypes - or crosses - which is helpful and necessary, but it is slow and very hard work to check it and an abbreviated form must also be provided in all figures for all data sets.

• We agree that the readers should be able to understand and interpret the figure in itself, yet we do not agree with the statement that we have not provided what is needed.

• First, abbreviated forms of the genotype were present in the figure legends whenever manageable (highlighted in cyan in the Tracked changes form to support our statement).
• In some occurrences with complex genotypes (Raeppli with dual genetic systems or CoinFLP data), we choose to explain the driver/transgene/conditions instead. For example, for Figure 3, the four full genotypes are:
tub-QS; Nrv2::GFP, wor-GAL4/CyO
x yw, hs-FLP; UAS-Raeppli-NLS 53D
x yw, hs-FLP; UAS-Raeppli-NLS 53D, QUAS-PTEN
x yw, hs-FLP; UAS-Raeppli-NLS 53D, QUAS-PTEN; UAS-shg RNAi^{VDRC27082}
In such case, our legend below (Previous figure legends lines 82-94) **stated as well as explained drivers, transgenes, markers and conditions**, which we think supported the interpretation of the figure:
"A) Schematic of the timing and genetic conditions used to probe the importance of NSC-driven timing for the encapsulation of individual NSC lineages. ***CG growth is initially blocked using PTEN expression in the CG through the use of the Q system (cyp-QF2 driver). At T1, the block is removed, and CG structure is assessed at T2.***
B) Representative confocal picture of the extent of individual encasing of NSC lineages by CG ***after (T2) the regimen described in A).*** *Top panel shows the whole thoracic VNC, and bottom panel a close-up of the yellow box.* ***NSC lineages were marked with the multicolour lineage tracing Raeppli-NLS (blue, white, orange and red), induced under the control of the GAL4/UAS system (wor-GAL4 driver) at ALH0 using hs-Flp. Larvae are dissected after 100 h at 29°C.*** *See Supplemental Table 1 for detailed genetics, timing, and conditions of larval rearing.* ***CG membrane is visualized with Nrv2::GFP (green).*** *Two representative CG chambers displaying individual encasing of NSC lineages are delineated with a white dashed line."*
• We have now added in the figure an abbreviated form describing the genotype, yet we have also keep a more functional description of the condition ("Control", "Delayed chamber formation", "Delayed chamber formation + *shg* knockdown") which seems to us more useful for the reader to understand what is the phenotypic output of the genetic manipulations.

• We also think we and Reviewer 3 have a different appreciation of what legends should be. We understand from Reviewer 3's comment that legends are expected to contain an explanation of how the experimental conditions were set up so that readers can assess if they were done correctly from a technical perspective. We do not think that is what the legends require. We agree that figures should be self-explanatory to readers, yet this does not always necessitate including technical details, but rather focusing on the outcomes of the techniques/conditions used. We prefer to keep legends simpler yet supporting interpretation (for example, what happens at T1 versus T2), while providing details in methods or supplemental for specialists (for example, how are T1 and T2 made).
• We have scanned again through the legends and added some details we felt would be relevant.

Terms must be defined before use: cell fate, cell identity and cell lineage are used interchangeably and in places it is ambiguous whether the authors refer to eg NSC/GMC/neuron cell fate or NSC1 vs NSC 2, neuron A vs neuron B, lineage/cell identity.

• We believe fate and identity are well-defined terms in biological research, bearing little controversy, and we did not used them interchangeably (we actually wrote "regardless of their identity/fate" at

Previous results Line 45, showing we do not consider them the same). They were also only used in the context of NSC-derived tumours (Figure 2).
• First, it was clearly stated which identity we were looking at:
Previous Results lines 3-4:
"CG encase only one, and not several NSCs within a membranous chamber, suggesting that **cell identity (*i.e.*, stem cell)** could be a cue for individual encasing."
And previous lines 21-23:
"These data show that both for Type I and Type II NSCs, **stem cell identity** is not sufficient to ensure their sorting into individual CG chambers."
• Similarly, fate was used in the context of cell transformation, as a mean to change cell identity. Fate leads to identity, which in turn can control identity further. In our case for example, expressing *pros* RNAi in NSC will change the fate of the progeny issued from asymmetric division (from differentiation to multipotent, less differentiated state), thus also altering their identity (from a GMC to a stem cell-like state).
We clearly stated it was meant this way, as a mean of transforming cell identity to test our hypothesis on the importance of stem cell identity:
Previous Results Lines 4-9:
"To assess the importance of NSC **fate** in chamber formation, we took advantage of genetic alterations known to dysregulate NSC division and differentiation and to lead to the formation of tumour-like, NSC-only, lineages [32]. In particular, *pros* knockdown in Type I lineages **converts GMC into NSC-like, Dpn^{+} cells at the expense of neurons** [33]."
• To remove any lingering doubt, we have now rephrased our text to remove the few occurrences of "fate" and only keep "identity" throughout.

• Regarding lineage, it was always with respect to the whole lineages of individual NSCs, so all the cell coming from one original NSC. We indeed clearly wrote NSC lineages. We have double-checked through the text to make sure the association was clear.
• We wrote once "neuronal lineage" in the abstract, as an introductory sentence stating the general question in the field. To remove any doubt, we have replaced with "newborn neurons".

To conclude, in the revised version, information on the experimental source of the evidence on which claims are grounded must be explicitly provided in the figures and figure legends for all data sets and improved within text.

• See our point above about legend contents.

2. Quantifications have improved greatly, which is excellent, but some are still missing (eg Supp Fig 3) - information must include how many samples they analysed and of these, how often they observed the phenotype (ie the penetrance), for all data sets. In some points, "penetrance 100%" is provided without sample size, which is meaningless.

• We have added quantifications for Supp. Fig. 3A (quantification of the percentage of non-encased NSC lineages at T1, now Supp. Fig. 3B), and Supp. Fig. 5B (quantification of the percentage of non-encased NSC lineages at T1, now Supp. Fig. 5C). Of note, we have also changed the representative picture of Supp. Fig. 3A to fit the mean of the corresponding quantification.

• We have looked through our legends and added the n for the panels corresponding to representative qualitative pictures, which were missing. We have highlighted in yellow the n numbers in the Tracked change text to confirm we have them for all data. As such, we think we can use the 100% penetrance stated in the Methods (we never had a sample within a batch showing no phenotype, only difference in expressivity).

In some places, interpretations lack rigour as they do not always accurately reflect the data. For example, authors state that "We then found that clonal tumour-like growth coming from single dysregulated NSCs (marked by the same colour) were contained within one CG chamber, both for Type I pros tumours and Type II brat tumours (Fig. 2C-D)." However, Supp Figure 2D wor>brat-RNAi shows that only 50% of clones contain one single colour. The authors interpret that Raepli did not work properly in the other 50% of cases. If so, Raepli data should not be included in the paper. Alternatively, if there are more Dpn+ NSC within a wor>brat-RNAi clone, Raepli should mark their lineages in different colours, as it did. This would mean that CG no longer distinguish lineages within a tumorous clone - the opposite interpretation to the authors'. Authors must clarify this. They must also modify their Interpretations consistently with the quantifications, regardless of their original narrative.

• We do not agree with this comment, and we do not try to stick to a narrative. We are confident that the differently-coloured cells within one CG chamber are not coming from different original mother NSCs following this reasoning:

1. As explained in the text, some uncontrolled Raeppli inductions happen in *brat* NSC-like Dpn$^+$ cells issued from one original mother NSC (amongst the 8 per CB). As such, all these cells, despite in some chambers being of different colours (not the majority as shown by the quantification of Supp. Fig. 2D), are from the same NSC lineage (again, as in coming from one of the 8 original mother NSCs).

2. These NSC-like Dpn$^+$ cells are labelled in a NSC lineage in which the original induction had not worked (unmarked lineage), after later spontaneous recombinations. This is supported by:

i) the detection of some clonal labelling without heatshock treatment;

ii) the observation that the chambers containing differently coloured cells are always containing non-coloured cells;

iii) the supernumerary count of coloured clones within one CB. If cells of different colors within a CG chamber came from different original mother NSCs, we would have expected no more than 8 distinct cell clones (marked by one colour) per CB. Yet, in CBs with multiple colours within one chamber, the total number of clones is always greater than 8 (see new Supp. Fig. 2F), while the number of Type II CG chambers is similar to control (see Supp. Fig. 2C). In the opposite case, and if some colours were found not separated by a CG membrane, we would have then concluded that CG group different *brat* lineages together.

• To summarize, the CG chambers containing differently coloured cells in *brat* tumour result from a technical artefact we have not been able to fully circumvent.

• To clear up this point, we now have added data for control and *brat* tumour with and without heatshock, including pictures and quantification of the sum of the number of colours across all Type II chambers per CB (Supp. Fig. 2 E-F).

• We are also providing new data using the CoinFLP technique to generate *brat* RNAi Type II clones and showing individual enclosure by the CG for 100% of such clones (Fig. 2E). We feel these data could be Supplemental, but we are running out of space (unless two supplemental figures per main figure are allowed).

• The text has also been remodelled (Results lines 45-59).

3. On the recommendation to authors to either provide evidence that 3xP3-GFP has been removed from the CRIMIC lines or remove the data and the claims, the authors did not act on these. Instead, the authors argued: "…We agree that the 3xP3-GFP of the CRIMIC lines is expressed in glial cells, at least partly in the CG. However, this cannot explain the nuclear, neat His::mRFP signal, which co-localises with Repo, a marker for glial nuclei (previous Supp. Fig. 5A) and thus which can only come from the CRIMIC enhancer…". I'm afraid this is invalid, these are not clean data as it is not possible to distinguish the origin of the glial signal. Stainings using CRIMIC lines that still carry 3xP3-GFP cannot be used in experiments on glial cells, because 3xP3GFP is very strong and visible throughout channels in

glia, including in nuclei. The data provided in Supplementary Figure 7D contain artefacts and must be removed, as well as the associated claim.

• There seems to be confusion regarding Supp. Figure 7D. Supp. Figure 7D. does NOT correspond to any CRIMIC staining, for which we did remove all the data. Instead, Supp. Figure 7D presented new data showing that the line we built containing both *cyp4g15-GAL4* and *worniu-GAL4* (the drivers we used separately all along) was driving (as expected) both in CG and in NSC lineages. Although proving that a genetically-built line contains what it should is (understandably) rarely provided when it comes from simply putting independent characterized transgenes together, we felt it was a welcome addition here since our data on *wrapper* and *neurexin-IV* interaction (Figure 7G-F) relies on the fact that this line indeed drives well in both cell populations.
• As such, considering the confusion about this figure's content, we think it should be kept as it is.

4. Statistics: On the request for p values for the data sets, authors argued: "All p-values were, and still are, displayed on the graphs themselves". The authors did provide p values for the post-hoc multiple comparisons tests, but they did not and still have provided the p values for the grouped data set tests in Figure 5C, Figure 7F, Figure 9F,I, Figure 10E, J, Figure 11D,E. For example, in Figure 11D, the figure has the p values of the post-hoc Dunn tests, but neither the figure nor the figure legend provide the p value of the Kruskal Wallis H test. For all data sets with more than 3 different sample types, the authors must provide the p values of the total data set (eg Kruskal Wallis Anova $p<0.001$ given either over figure or in figure legend next to the name of the test applied, and post-hoc multiple comparisons Dunn test *$p<0.05$ provided in figure graph). Also, standard test names should be provided (eg Unpaired Student t test; Mann Whitney U test).

• We now understand what was asked. We did not add the group data statistics indicating there was any difference (or not) within the whole set, as we thought individual comparisons would be what is informative for the reader. We also did not think/know it was standard practice to systematically add such value in addition to the post-hoc values.
• We have now added these values in the figure legends, as we found it could possibly bring confusion on the figure itself. For some (*e.g.*, Figure 9F), it was not possible as some samples have no variance.

Other:

Figure 1 should be discussed within Results, not Introduction.

• We do not think it is unusual in a paper to have an introductory figure to help the reader grasping its context, starting from a summary of what is known from previous studies. Figure 1 does not contain new results from our study, but rather schematics or imaging data used as supportive/summary illustrations.
• For now, we have mostly kept it as it was, as we felt moving the description of the CG chamber out of the introduction does not set the stage properly. We have however moved the data on *worniu-GAL* expression (Supp. Fig. 1B-C) at the beginning of the results, as we characterized it within the CG context in this study.

Shg LOF: authors argued: "… we backed up these data using null shg mutant clones (in which the loss of Shg signal was also checked for extra-safety, previous Supp. Fig. 3F, now Supp. Fig. 55A), and this genetic approach cannot fit the issue with the RNAi not working." However, Supp Fig 5A shows shg knock-down, not mutant clones.

• This was a mistake in figure numbering in the Response to reviewer, which we apologize for. Nevertheless, that does not change the fact that *shg* null mutant clones (clearly present and discussed

in the paper) are not fitting the issue with the RNAi not working. We struggle to understand the point made here.

Abstract: "corset"? is not a helpful metaphor

• We have replaced it by “belt”.

"We expressed…PTEN" should be over-expressed.

• We have changed the text accordingly/

Figure 7F: "We then used the same approach to assess NSC encasing upon double knockdown of nrx-IV and wrapper ….. These data pinpoint an additive effect of nrx-IV and wrapper knockdowns …". This is incorrect. If the phenotype of the double knock-down is stronger than those of the single knock-downs, then there is a synergistic phenotype - not additive. Synergistic (not additive) phenotypes reveal a genetic interaction; additive phenotypes mean the genes do not interact.

• We agree we should not have put additive (in which case indeed we should expect the double knockdown to correspond to the added effect of the independent knockdowns). The meaning we had in mind was “additional” (*i.e.*, added, supplemental effect compared to a single knockdown), I am afraid I did not know there was a term distinction in English and I am really sorry for this mistake.

• However, we do not feel comfortable writing synergistic. The definition of synergy is not as steady as other genetic interactions. Reviewer 3 defines synergy as when the double condition displays an effect greater than each of the independent condition. Yet, it is also defined as when the combined impact on the phenotype resulting from both conditions is greater than the sum of their individual effects. See for example PMID: 19665253 for a recent review on synergy in genetics. This is the definition I was more familiar with (synergy gives you more that what you could predict from the independent effects, something “new”), and I do not think that is what is seen in our experiment.

• As such, we propose to stay more neutral in defining *nrx-IV* and *wrapper* interaction. The fact that the phenotype of one condition is influenced by the another is clear and allows to conclude for a genetic/epistatic interaction between them (*i.e.,* straying from independence), which we believe is sufficient for the study.
• We have remodeled the sentence as follows (Results lines 525-529):
“These data pinpoint a greater effect of the combined nrx-IV and wrapper knockdowns than their individual contribution on NSC lineage encasing by the CG, showing that the genotype of one affects the phenotype of the other. This demonstrates an epistatic interaction between nrx-IV and wrapper in this context.”

The manuscript and its future impact would greatly benefit from being much more concise.

• We understand this point and have try to shorten specific paragraphs which felt the heaviest. Considering the number of main/supplementary figures (24 full figures) and associated data, and the technical details we were asked to provide in the text, we found it difficult to go down much more. We have highlighted in grey in the Tracked change file some paragraphs which could be removed without affecting the meaning of the paper, yet we feel it would be a loss for discussion. We welcome the editors’ feedback on this matter.
